# Fourier transform infrared spectroscopy enables rapid strain typing and cluster analysis of *Listeria monocytogenes* under diverse growth conditions

**DOI:** 10.3389/fmicb.2026.1735218

**Published:** 2026-02-17

**Authors:** Francis Muchaamba, Marc J. A. Stevens, Roger Stephan

**Affiliations:** Vetsuisse Faculty, Institute for Food Safety and Hygiene, University of Zürich, Zurich, Switzerland

**Keywords:** cluster analysis, foodborne pathogens, Fourier-transform infrared spectroscopy (FTIR), *Listeria monocytogenes*, microbial strain typing, real-time outbreak investigation

## Abstract

Fourier-transform infrared (FTIR) spectroscopy offers a rapid, high-throughput method for microbial strain typing, with potential applications in food safety and outbreak investigations. We evaluated its discriminatory power for typing *Listeria monocytogenes* using 118 strains representing diverse serotypes. FTIR showed high reproducibility, with tight clustering of spectral profiles from technical and biological replicates across multiple days, supporting its utility as a dereplication tool. Both hierarchical clustering and an artificial neural network classifier achieved 100% accuracy at the serogroup level, with an adjusted Rand index of 1.00 compared to conventional serotyping. However, FTIR lacked the resolution of whole-genome sequencing (WGS), likely due to spectral overlaps in polysaccharide-associated regions among clonal complexes and some serotypes (e.g., 1/2a, 1/2b, 1/2c). Despite this, FTIR could differentiate related and unrelated isolates in outbreak or persistence strain cluster analyses. Spectral profiles were significantly influenced by the bacteria growth conditions, with the strongest clustering coherence observed among isolates cultured on the same media. ALOA, Blood, and Oxford agar yielded the best classifier performance, whereas RAPID’L.mono produced the lowest scores. Incubation temperature and duration also influenced spectral quality and classification confidence. Lower temperatures (25 °C vs. 37 °C) enhancing serotype-level discrimination within serogroups, highlighting the importance of optimized culture protocols in improving the discriminatory power of FTIR-based strain typing. Overall, our data supports the use of FTIR as a rapid initial screening method for pathogen typing and persistence or outbreak-related cluster detection, helping prioritize isolates for confirmatory WGS. Expanding classifier databases and integrating FTIR with molecular methods could further improve its resolution and applicability for real-time pathogen typing.

## 1 Introduction

Ensuring food safety has become increasingly challenging due to the rapid industrialization of food production, the impact of global warming, and shifts in consumer preferences towards organic, minimally processed, and ready-to-eat (RTE) foods (Vlachos and Georgantzis, 2016; Wambogo et al., 2020; Duchenne-Moutien and Neetoo, 2021; Meijer et al., 2021). These challenges are further compounded by an increasing population of vulnerable individuals and the emergence of enhanced-virulent, antimicrobial-resistant foodborne pathogens (Kim and Ahn, 2022; Duma et al., 2024; Hu et al., 2024). Consequently, there is an urgent need for efficient and rapid methods to analyse food and ensure its safety.

Among foodborne pathogens, *Listeria monocytogenes* poses a significant public health threat, with infections often linked to contaminated RTE foods. Infections can lead to severe morbidity and mortality, particularly among high-risk populations such as pregnant women, neonates, and immunocompromised individuals (Buchanan et al., 2017). The persistence of *L. monocytogenes* in food-processing environments is facilitated by its stress tolerance and ability to form biofilms, which help it to withstand standard cleaning and disinfection protocols (Duze et al., 2021; Fagerlund et al., 2021; Osek et al., 2022; Finn et al., 2023). Additionally, its ubiquitous distribution makes it easy to be introduced into production facilities, where it can establish long-term contamination reservoirs.

*Listeria monocytogenes* isolates are traditionally differentiated into four serogroups (1/2, 3, 4, and 7), which are further subdivided into 14 serotypes, 1/2a, 1/2b, 1/2c, 3a, 3b, 3c, 4a, 4b, 4c, 4d, 4e, 4h, and 7, based on variations in somatic O and flagellar H antigens (Maury et al., 2016; Yin et al., 2019). Notably, serotypes 1/2a, 1/2b, and 4b account for 95% of human listeriosis cases (Orsi et al., 2011), with serotype 4b most frequently implicated in outbreaks and clinical cases probably due to its increased virulence (Maury et al., 2016; Muchaamba et al., 2021). Molecular typing techniques, including PCR and multi-locus sequence typing (MLST), have enabled the classification of isolates into PCR serogroups [e.g., IIa (1/2a, 3a), IIb (1/2b, 3b, 7), IIc (1/2c, 3c), IVb (4b, 4d, 4e), IVb var. (4b), and L (4a, 4c)], and the identification of clonal complexes (CCs) (Doumith et al., 2004; Ruppitsch et al., 2015). These CCs and serotypes have been grouped into four major lineages (I–IV), which are associated with distinct virulence, environmental distribution, and host range (Hyden et al., 2016). For instance, strains of serotype 4b and 4h, have been shown to be more virulent compared to other serotypes (Yin et al., 2019; Muchaamba et al., 2021). Timely and accurate detection and typing of such pathogens is crucial for preventing outbreaks, mitigating their impact, and controlling infections. It is also essential for monitoring *L. monocytogenes* in processing plants, enabling risk-based inspection programs, and ensuring effective decontamination to prevent contamination of end products. As the relationship between serotype, MLST, and pathogenic potential becomes clearer, the call for risk-based regulation of *L. monocytogenes* intensifies, underscoring the need for fast and accurate strain typing.

However, routine analysis of *L. monocytogenes* by traditional serotyping methods, such as agglutination assays, is limited by labour intensity, cost, and the need for technical expertise, with reproducibility at times being challenging (Doumith et al., 2004; Gorski, 2014). PCR-based techniques offer faster and more cost-effective alternatives; however, they still require trained personnel and may lack the resolution needed for detailed strain-level differentiation. These limitations can reduce their effectiveness for real-time surveillance and outbreak response. Advanced molecular approaches like MLST and whole genome sequencing (WGS) provide high-resolution insights but are constrained by higher costs, longer turnaround times, and the need for specialized bioinformatics expertise (Ruppitsch, 2016). Nevertheless, recent advances in sequencing technologies, such as Oxford Nanopore coupled with artificial intelligence (AI), are helping to reduce turnaround times and make WGS more accessible for routine use (Urban et al., 2023). Similarly, matrix-assisted laser desorption/ionization-time of flight (MALDI-TOF) mass spectrometry enables rapid species identification but lacks the discriminatory power required for accurate strain or serotype differentiation (Gant and Chamot-Rooke, 2024).

Fourier-transform infrared (FTIR) spectroscopy has emerged as a promising alternative for rapid, cost-effective pathogen typing (Al-Fraihat et al., 2025; Lurie-Weinberger et al., 2025). By measuring the absorption of infrared light at specific wavelengths, FTIR detects vibrational energy transitions of molecular bonds in cellular micro- and macromolecules, generating unique “spectral fingerprints” that reflect the biochemical composition of cells (Rakovitsky et al., 2020; Muchaamba and Stephan, 2024). This technique has demonstrated comparable performance to WGS in specific outbreak investigations, such as tracking multidrug-resistant *Acinetobacter baumannii* (Lombardo et al., 2021) and differentiating extended-spectrum *β*-lactamase-producing *Klebsiella pneumoniae* strains (Rakovitsky et al., 2020). Despite its potential, challenges such as inter-laboratory reproducibility and species-specific limitations (e.g., with *Staphylococcus aureus*) necessitate further optimization and validation of FTIR protocols.

The integration of FTIR with specialized analysis software, such as in the IR Biotyper® system, presents an opportunity to enhance pathogen classification processes. By leveraging advanced spectral analysis capabilities alongside AI and machine learning, FTIR’s discriminatory power is significantly enhanced, facilitating fast strain typing. The global nature of foodborne pathogen outbreaks highlights the need for widely accessible strain classification databases to standardize pathogen tracking and outbreak investigations, much like MALDI-TOF MS has done for species identification. Existing FTIR databases for *L. monocytogenes* (Oberreuter et al., 2023), *Salmonella* spp. (Cordovana et al., 2022; Oberreuter et al., 2025), *Streptococcus pneumoniae* (Passaris et al., 2022), and *Legionella pneumophila* (Pascale et al., 2022) demonstrate the potential of this approach.

Various FTIR systems, including those combined with multivariate analysis or machine learning algorithms like Artificial Neural Networks (ANN), have been successfully applied to differentiate *Listeria* species and *L. monocytogenes* serogroups, serotypes, or haplotypes, with reported accuracies of 80%–100% (Rebuffo et al., 2006; Rebuffo-Scheer et al., 2007; Janbu et al., 2008; Davis and Mauer, 2011; Romanolo et al., 2015; Oberreuter et al., 2023). However, despite these promising results, most previous studies were conducted under conditions that limit FTIRs direct applicability to routine food testing and outbreak response workflows. In particular, the use of non-selective growth media such as Blood agar (Oberreuter et al., 2023), Tryptone soy agar (TSA) (Rebuffo et al., 2006), and Tryptic soy broth (TSB) (Romanolo et al., 2015) does not align with ISO 11290-1:2017 recommendations, which specify selective chromogenic media for *Listeria* spp. isolation. As a result, additional subculturing steps are often required to meet FTIR spectral quality standards, delaying analytical workflows and reducing compatibility with routine diagnostic protocols. Moreover, key performance characteristics, including reproducibility over time, robustness to variation in growth conditions, discriminatory power at different typing levels, and database coverage across serogroups, serotypes, and clonal complexes, have not been systematically evaluated within a single, standardized framework. Many studies have also relied on custom-built data analysis pipelines, lacking integration into standardized, user-friendly platforms, further hindering broader adoption in diagnostic and regulatory settings. Collectively, these limitations highlight the absence of a clear benchmarking framework for evaluating FTIR performance under ISO-relevant culture conditions and in relation to operational needs, particularly when compared to WGS-based workflows. Consequently, the practical added value of FTIR in the current era of increasingly routine WGS remains insufficiently defined, particularly for early-stage triage, dereplication, and prioritization of isolates. This is especially relevant in settings where sequencing capacity, turnaround time, bioinformatic processing, and data management can still constrain the rapid analysis of large isolate collections.

In this study, we address these gaps by evaluating the discriminatory power of FTIR for distinguishing *L. monocytogenes* serogroups and strain clusters using the IR Biotyper® system, a commercially available platform. We benchmarked its performance against traditional typing methods and systematically assessed the impact of culture media and sample preparation on FTIR performance. We also evaluate a pre-developed FTIR-ANN-based serogrouping classifier using a diverse set of well-characterized field strains, testing its resolution and accuracy. In addition, we examine FTIR’s utility for outbreak or persistence investigation and dereplication (i.e., identification of redundant / duplicate isolates), with the aim of defining its role as a rapid first-line screening tool complementary to WGS.

## 2 Materials and methods

### 2.1 Study strains

A total of 118 *L. monocytogenes* strains, representing a diverse range of serotypes, MLST CCs, and genetic backgrounds (lineages and sequence types), were included in the analysis (Table 1; Supplementary Tables S1, S2). Whenever possible, at least five strains per serotype were selected. The selected serotypes are commonly associated with clinical cases and food contamination. Data on WGS, MLST CCs, and serotyping (via agglutination reactions with *L. monocytogenes* antisera from Denka Seiken Co., Japan), are available for most isolates, except for seven strains that lack WGS data. The strains included in this study cover 26 distinct *L. monocytogenes* MLST CCs (Supplementary Figure S1). All isolates were stored at −80 °C in Brain Heart Infusion (BHI) broth (Oxoid) with 20% glycerol (Sigma-Aldrich). Importantly, these isolates were not part of the strain sets previously used for the development of the ANN-based classifier by Bruker or in other earlier studies (Rebuffo-Scheer et al., 2007; Romanolo et al., 2015; Oberreuter et al., 2023).

**Table 1:** Overview of study strains.

Serotype	Number of strains
1/2a	50*
1/2b	10
1/2c	12
3a	7
3c	3
4b	32
4 a/c	4

*Includes 15 isolates only used in the blinded cluster analysis study.

### 2.2 Strain cultivation and sample preparation for FTIR analysis

A total of 103 *L. monocytogenes* strains were revived from −80 °C cryo stocks by two successive subcultures (primary and secondary cultures) on Columbia Sheep Blood agar (Blood agar; Oxoid), and BHI agar (Oxoid). These cultures were used to assess the discriminatory capacity of FTIR spectroscopy at both the serogroup and strain levels. To evaluate the influence of growth media on FTIR profiles, a subset of 32 isolates, representing diverse serotypes and genetic backgrounds (1/2a, 1/2b, 1/2c, 3a, 3c, 4a/c, and 4b; Supplementary Table S1), was selected. These isolates were revived by subculturing twice on ALOA (Bio-Rad), Palcam (Merck), RAPID’L.mono (Bio-Rad), TSA (Oxoid), or *Listeria* Selective Media (Oxford agar; Oxoid). All plates were incubated aerobically at 37 °C for 24 h. For broth cultures, single colonies from primary BHI agar plates (from the same subset of 32 isolates), were inoculated into 10 mL BHI broth and incubated at 37 °C with shaking at 150 rpm for 24 h.

To assess the influence of incubation temperature and duration on FTIR-based strain differentiation, the subset of 32 *L. monocytogenes* strains was cultured under varying conditions before FTIR analysis. Single colonies from primary Blood agar, ALOA, Oxford, and Rapid’Lmono agar plates were subcultured onto corresponding agar plates and incubated at 25 °C for 24 h to simulate agglutination serotyping culture conditions. A subset of 15 isolates was also subcultured on either Blood agar or ALOA agar and incubated at 37 °C for 20 h or 48 h. The resulting cultures were subsequently subjected to FTIR analysis.

For agar-grown samples, a 1 μL loop of microbial colony material was collected from the confluent region of the plate (avoiding agar contamination) and resuspended in 50 μL of 70% ethanol in suspension vials containing metal rods (IR Biotyper® kit). The suspension was vortexed, then 50 μL of molecular-grade water was added. For broth-grown samples, cells were harvested by centrifuging 7 mL of culture at 6400 × *g* for 5 min at 20 °C. The resulting pellet was washed twice with sterile normal saline (0.8% NaCl) and resuspended in 100 μL of molecular-grade water. An equal volume (100 μL) of 70% ethanol was added, followed by vortexing to ensure homogenization. For both sample types, 15 μL of the prepared suspension was spotted in quadruplicate onto a 96-well silicon IR Biotyper® target plate. Quality control standards (IRTS 1 and IRTS 2) were prepared according to the IR Biotyper® kit instructions and included in duplicate on the target plate. The plate was dried at 37 °C for 20–30 min, ensuring that all sample spots were visibly dry but not cracked. A blank well was used for background measurement.

### 2.3 Spectral acquisition and analysis

FTIR analysis was performed using the IR Biotyper® (Bruker Daltonics GmbH & Co. KG) in transmission mode with OPUS 8.2 software (Bruker Optics), following the manufacturer’s protocol. Absorption spectra were recorded from 4,000 to 500 cm^−1^ with 32 scans per sample, intercalating the background well spectra for correction. After acquisition, spectra underwent quality assessment, and those with suboptimal quality were excluded from further analysis. Accepted spectra were processed in the IR Biotyper® Client software V4.0 (Bruker Daltonics), applying second derivative calculation, relevant spectral region splicing, and vector normalization. Each strain was analysed with a minimum of two biological replicates, with each three technical replicates. Data analysis primarily focused on the default spectral window (1,300–800 cm^−1^), which corresponds to carbohydrates/polysaccharides. Additional spectral windows, corresponding to lipids, proteins, or other customized regions, were evaluated as needed. Spectral windows used by Rebuffo et al. (2006) (3,100 to 2,800 cm^−1^, 1,800 to 1,500 cm^−1^, and 1,200 to 700 cm^−1^) and Rebuffo-Scheer et al. (2007) (1,200 to 900 cm^−1^ and 1,800 to 1,400 cm^−1^) were also analysed. Principal Component Analysis (PCA) and Linear Discriminant Analysis (LDA) were applied for dimensionality reduction, and results were visualized as dendrograms, distance matrices, and scatter plots to illustrate strain clustering and differentiation. Additionally, all spectra were classified based on the 1,300–800 cm^−1^ spectral window using the manufacturer’s commercially available *Lmono* serogrouper classifier (Bruker Daltonics), integrated within the IR Biotyper® Client software. This classifier is based on an ANN trained to differentiate *L. monocytogenes* isolates into four serogroups, Serogroup 1/2 (serotypes 1/2a, 1/2b, 1/2c), Serogroup 3 (3a, 3b, 3c), Serogroup 4 (4a, 4b, 4c, 4d, 4e), and Serogroup 7 (7). The ANN uses the first 30 principal components derived from second-derivative, vector-normalized FTIR spectra within the 1,300–800 cm^−1^ region and was trained using 200 training cycles (Bruker Daltonics, I.R. Biotyper Software User Manual, 2022). This spectral window was preselected by the manufacturer as optimal for serogroup discrimination. The classifier was trained with spectra from 82 strains grown at 37 °C for 24h on all the media tested in this study except BHI broth.

The serogroup classification results for each spectrum were compared to the original serological serotype assignment for each strain, and any discrepancies were noted. Classification scores, which reflect the confidence levels in FTIR serogroup assignment, were recorded and evaluated across different media and incubation conditions. The scores are color-coded, green (<1.0) for spectra within training set boundaries, considered highly reliable, yellow (1.0–2.0) for spectra near boundaries considered moderately reliable, and red (>2.0) for spectra outside training set boundaries, deemed unreliable and unclassified. A spectrum was considered misclassified if it was assigned an incorrect serogroup with a green or yellow classification score.

#### 2.3.1 Spectral analysis of growth condition variations

To visualize and compare spectral variations, an average spectrum was generated for representative strains using OPUS 8.2 software. For each growth condition, at least two biological repeats, each with three technical replicates, were averaged. The resulting spectra were systematically analysed across strains, serotypes, and growth conditions to identify spectral regions most affected by variations in growth conditions.

#### 2.3.2 Evaluation of FTIR reproducibility

To assess FTIR reproducibility, four *L. monocytogenes* strains (EGDe, 10403S, LL195, and LMNC318) were selected. For each strain, colonies were independently cultured on Blood agar and BHI agar at 37 °C for 24 h on four separate days, with three technical replicates analysed per each FTIR setup. The resulting spectra were evaluated against a defined cut-off value to evaluate consistency. Clustering was deemed reproducible if all biological and technical replicates for each strain grouped within the same cluster, without separation between biological replicates.

### 2.4 Comparison of FTIR and other typing methods

The similarity between clusters generated by FTIR, PCR-based typing, serological serotyping, MLST, and WGS was assessed using three metrics, Simpson’s Index of Diversity (SID), Adjusted Rand Index (ARI), and Adjusted Wallace Coefficient (AWC). These metrics, along with their 95% confidence intervals (CIs), were calculated using the online tool Comparing Partitions^1^ (Carriço et al., 2006). SID was used to evaluate the diversity of clusters identified by each method. ARI measured the agreement between clustering methods, adjusting for random chance, with values closer to 1 indicating higher concordance (Carriço et al., 2006; Pinto et al., 2008; Severiano et al., 2011b; Severiano et al., 2011a). The AWC assessed directional congruence, quantifying the probability that isolates clustered together by one method (e.g., WGS) were also clustered together by FTIR, and vice versa. A value of 1 represents complete congruence. For all analyses, WGS was considered the gold standard.

### 2.5 Blinded study to determine applicability of FTIR for strain cluster analysis

To evaluate the potential of FTIR for real-time strain cluster analysis simulating outbreak or persistence scenarios, a blinded study was conducted. The investigator performing the FTIR analysis was unaware of both the number of strain clusters (as determined by cgMLST) and the number of isolates per cluster included in the study. A total of 15 isolates were analysed, consisting of 10 isolates from three cgMLST based clusters and 5 epidemiologically unrelated outgroup isolates, all belonging to the same MLST CC (Supplementary Table S2). These isolates were analysed separately from the initial set of 103 strains described earlier in the study.

To maintain blinding, the identity of each isolate was withheld from the investigator until the FTIR analysis and conclusions were finalized. The isolates were revived from −80 °C cryo stocks through two successive subcultures on either Blood agar or Rapid’Lmono agar, incubated at 37 °C for 24 h, and subsequently analysed using FTIR. The resulting spectra were analysed as described above within the IR Biotyper® Client software v4.0. After unblinding, clustering accuracy was evaluated by cross-referencing the FTIR results with WGS-based phylogenetic analyses using ARI and AWC.

#### 2.5.1 Genome analysis

To assess the genetic relatedness of isolates in the blinded study, cgMLST analysis was performed following the previously defined core genome scheme (Ruppitsch et al., 2015). Whole-genome sequencing of these isolates was conducted in earlier studies using the Illumina MiniSeq platform (Illumina, San Diego, CA, United States), as previously described (Stevens et al., 2024). This Whole Genome Shotgun project has been deposited at DDBJ/ENA/GenBank under the accession JBNVUV000000000 to JBNVVJ000000000. Each genome (Supplementary Table S2) was blasted against a set of 1,701 loci, and cluster types were assigned using Ridom Seqsphere+ v9.0.1 (Ridom GmbH) with default parameters. Missing genes were disregarded in all analyses. Minimum spanning trees were generated in SeqSphere+ to visualize strain relationships, with a cluster defined as isolates differing by ≤10 alleles (Ruppitsch et al., 2015).

## 3 Results

### 3.1 Reproducibility and discriminatory power of FTIR for *Listeria monocytogenes* strain typing

**Figure 1:**
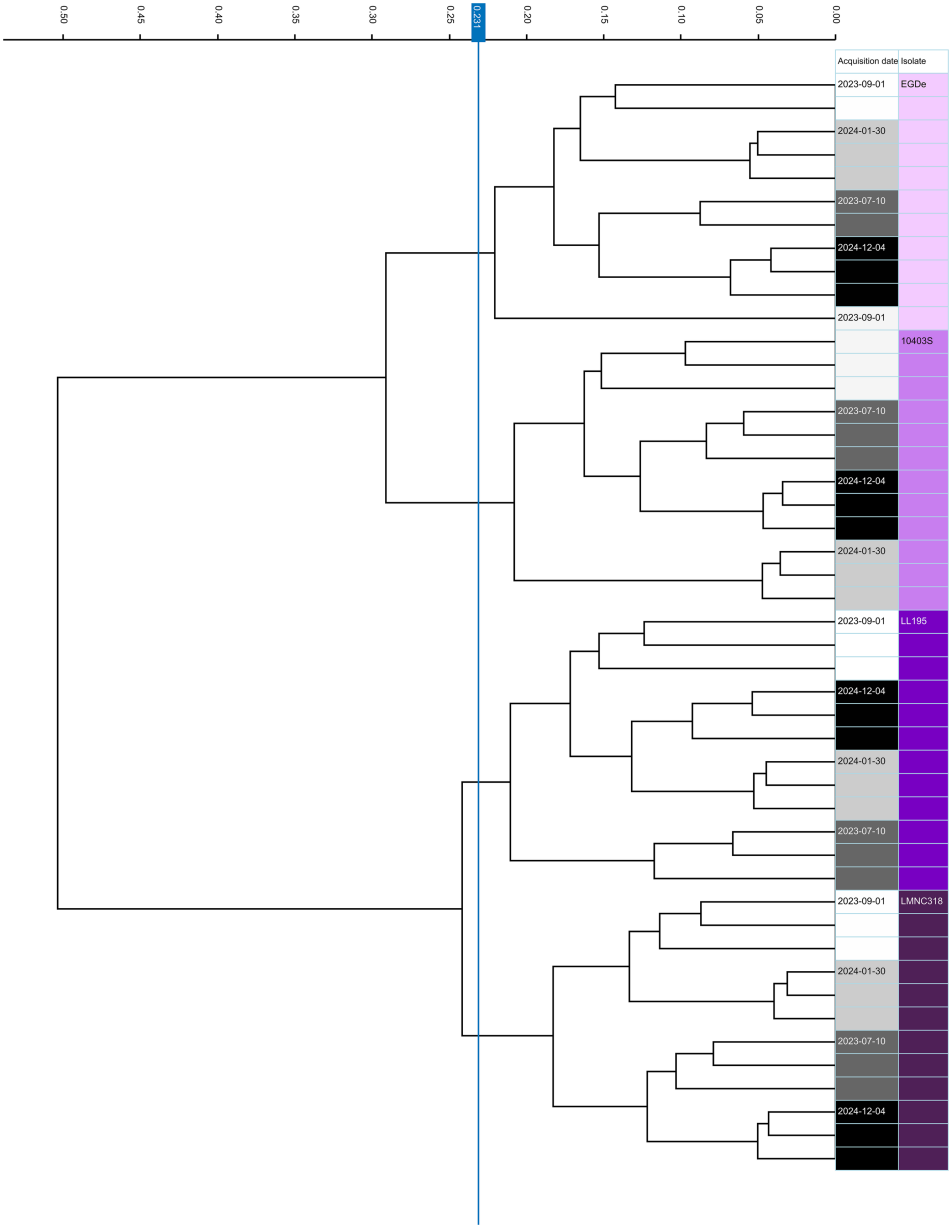
FTIR-based clustering and reproducibility across replicates. Dendrogram illustrating the clustering of FTIR spectra from *Listeria monocytogenes* strains EGDe, 10403S, LL195, and LMNC318 to assess strain differentiation and reproducibility across biological and technical replicates. Strains are color-coded by strain ID and acquisition date. Rows represent technical replicates derived from four independent experiments using cultures grown on blood agar at 37 °C on four separate days, spanning 513 days between the first and last measurements. The vertical blue line indicates the automatically calculated clustering cut-off value (0.231). No dimensionality reduction was applied. Analysis was based on the 1,300–800 cm^−1^ spectral window.

To assess the reproducibility of FTIR, a long-term analysis was conducted on four *L. monocytogenes* isolates representing serotypes 1/2a, 1/2b, 4a/c, and 4b, across four different days, with three technical replicates per strain. The first and last measurements were taken 513 days apart, allowing evaluation of reproducibility over an extended period. Both biological and technical replicates formed tight clusters, effectively grouping spectra by strain and demonstrating the reproducibility and reliability of the method (Figure 1). This high reproducibility was consistent across all isolates throughout the study.

**Figure 2:**
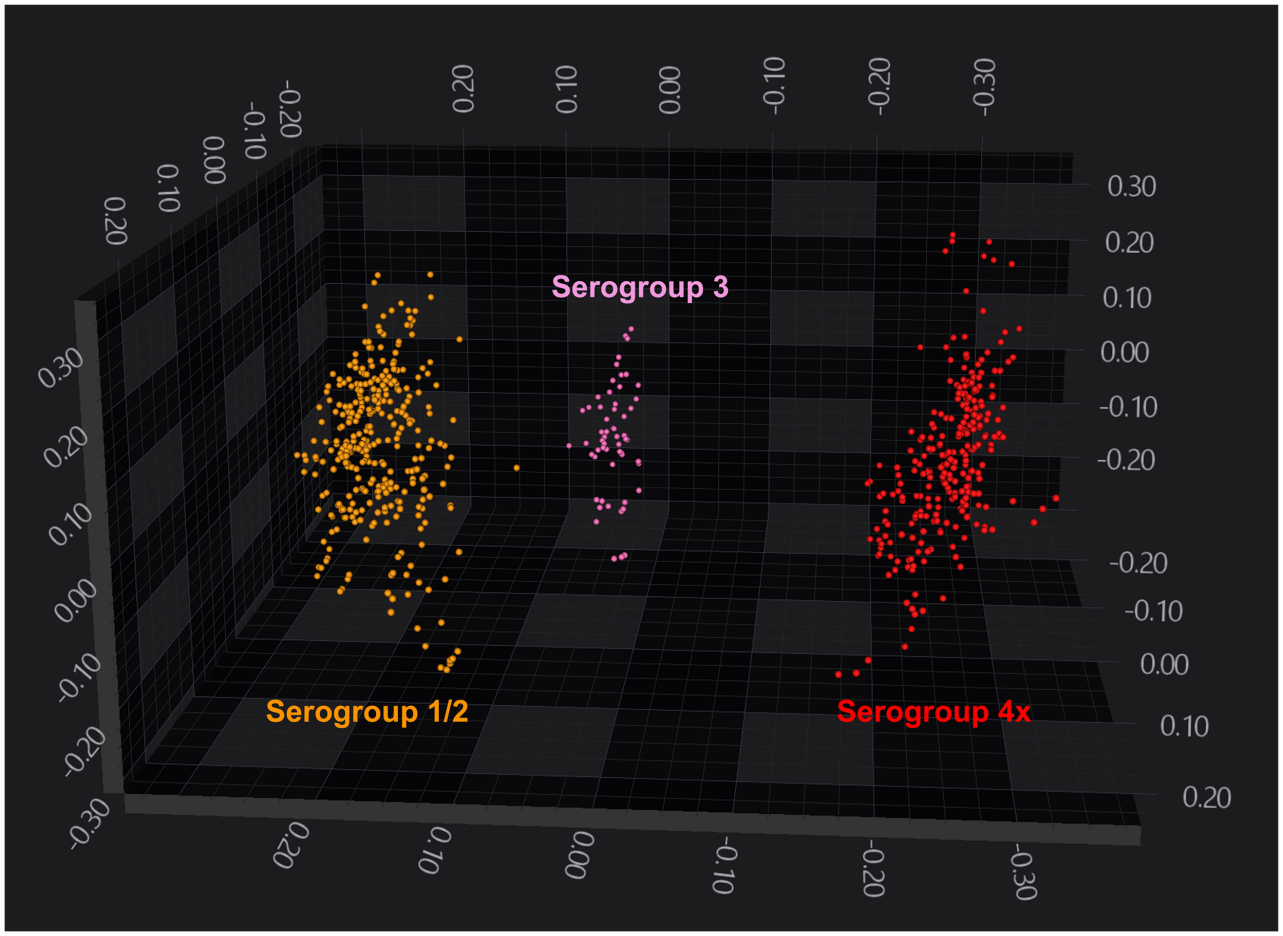
FTIR-based clustering of *Listeria monocytogenes* strains by serogroup. 3D scatter plot showing the distribution of *Listeria monocytogenes* strains in the FTIR spectral space. The plot displays the first three principal components (PCs) axes. Spectra are color-coded by serogroup (orange: 1/2: pink: 3, and red: 4) and each symbol (•) represents a technical replicate derived from at least two independent biological replicates per strain. The analysis was based on the 1,300–800 cm^−1^ spectral window. Dimensionality reduction was performed using PCA with 11 PCs, capturing 95% of the variance.

**Figure 3:**
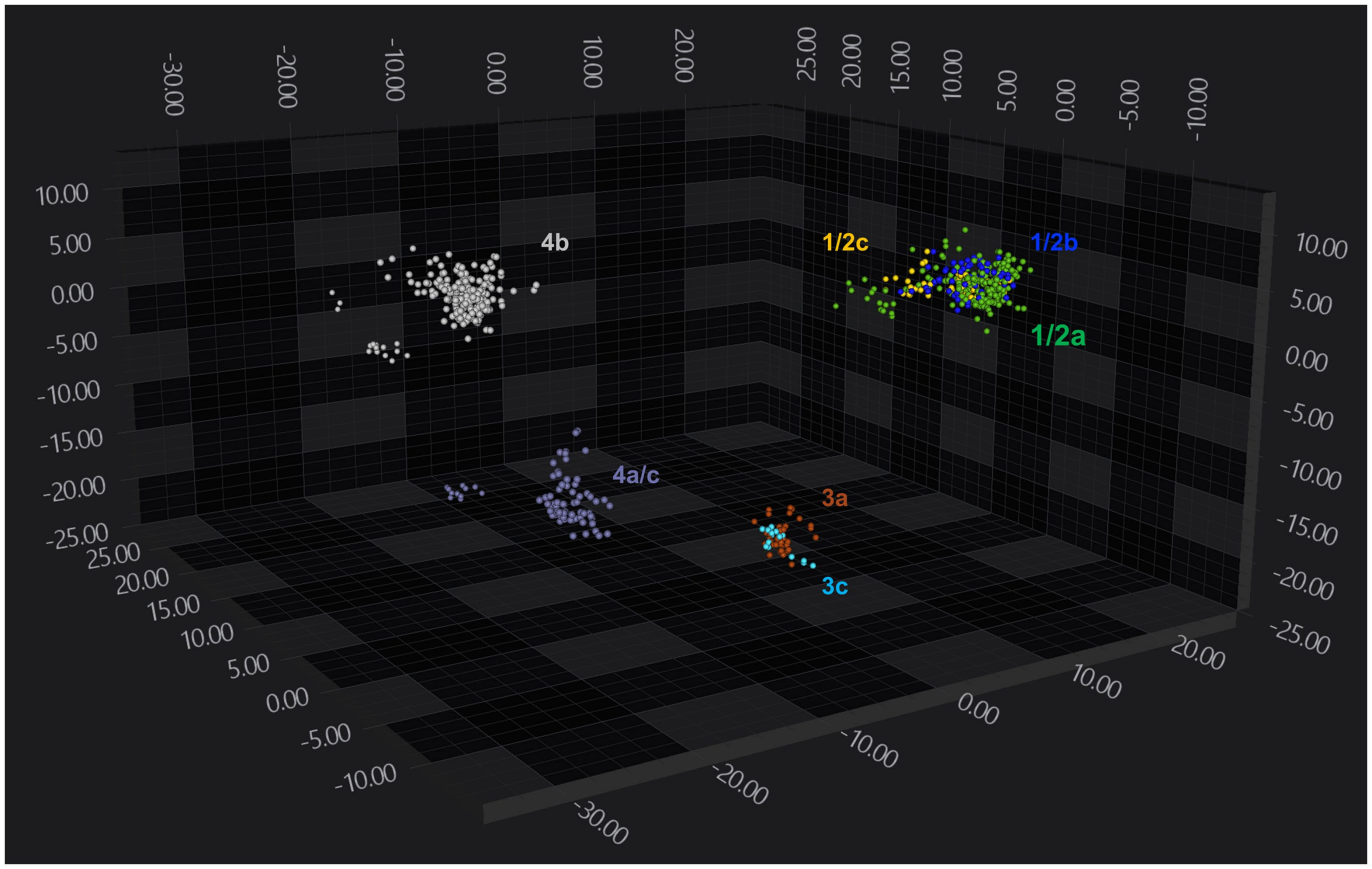
FTIR-based clustering of *Listeria monocytogenes* strains by serotype. 3D scatter plot showing the distribution of *Listeria monocytogenes* strains within the FTIR spectral space. The plot displays the first three LD axes and spectra are color-coded by serotype (green: 1/2a; blue: 1/2b; yellow: 1/2c; brown: 3a; cyan: 3c; purple: 4a/c; grey: 4b). Each symbol (•) represents a technical replicate derived from at least two independent biological replicates per strain. All cultures were grown on Blood agar 37 °C for 24 h. The analysis was based on the 1300–800 cm^−1^ spectral window. Dimensionality reduction was performed using LDA with 11 PCs, capturing 95.5% of the variance.

Analysis of 103 strains grown on either Blood agar or BHI agar demonstrated that FTIR-based clustering efficiently discriminated between *L. monocytogenes* strains. FTIR achieved 100% accuracy in distinguishing broad *L. monocytogenes* serogroups 1/2, 3, and 4 (Figure 2), indicating strong discriminatory power at the serogroup level. However, FTIR did not capture fine-scale genotypic differences reflected in MLST (Supplementary Figure S2), highlighting its potential role as a phenotypic first-pass tool, with complementary genetic methods used for more detailed high-resolution analysis. Additionally, it could not reliably distinguish some serotypes. For instance, serotypes 1/2a, 1/2b, and 1/2c clustered together, forming a single serogroup 1/2 cluster, likely due to spectral overlap in key polysaccharide regions. Notably, when LDA dimensionality reduction was applied, using isolate identity as the target label, FTIR successfully distinguished serotype 4b isolates, which formed a distinct cluster separate from other serogroup 4 strains (Figure 3; Supplementary Figure S3).

### 3.2 Assessment of clustering efficiency using different spectral windows

To further evaluate FTIR clustering performance, we explored strain differentiation using alternative spectral windows. Spectral windows such as 1,200–900 cm^−1^, along with combined regions “3,100–2,800 cm^−1^, 1,800–1,500 cm^−1^, and 1,200–700 cm^−1^”, as well as “1,200–900 cm^−1^ and 1,800–1,400 cm^−1^”, achieved 100% accuracy in strain- and serogroup-level clustering, similar to the default spectral window (1,300–800 cm^−1^) (Supplementary Figure S4). These results confirm that serogroup classification models can be developed using these alternative spectral windows as well. When applying LDA dimensionality reduction, these alternative windows successfully distinguished serotype 4b isolates from other serogroup 4 strains, as seen with the default 1,300–800 cm^−1^ window (Figure 3; Supplementary Figure S5). However, none of the alternative windows improved resolution at the serotype or MLST level. They failed to differentiate closely related serotypes within serogroups 1/2 and 3, indicating that spectral overlap among these serotypes persists across these regions as well.

### 3.3 Assessment of the FTIR-based *Listeria monocytogenes* serotype classifier

Next, we assessed the performance of the IR Biotyper® manufacturer’s *Lmono* serogroup classifier using cultures grown on Blood agar and BHI agar. The ANN-based classifier accurately identified the serogroups of all study strains (Supplementary Table S1), with FTIR-based typing into broad serogroups (1/2, 3, and 4) showing perfect agreement with serological serotyping results (ARI = 1.00). Specifically, strains from serotypes 4a, 4b, and 4c were assigned to serogroup 4x, serotypes 3a and 3c to serogroup 3, and serotypes 1/2a, 1/2b, and 1/2c into serogroup 1/2, according to the current FTIR database classification. Interestingly, the confidence of serogroup predictions varied between spectra from the same strain, depending on the growth medium. Cultures grown on Blood agar generally yielded higher confidence scores, with 93.6% (799/854) of spectra having green scores (<1.0), indicating that the spectra were within the classifier’s training set boundaries and considered reliable (Supplementary Table S3). In contrast, spectra from BHI agar grown cultures showed a slightly lower proportion of green scores, with 92.8% (798/860) falling within this range.

### 3.4 Influence of growth conditions and strain preparation on FTIR-based strain typing

**Figure 4:**
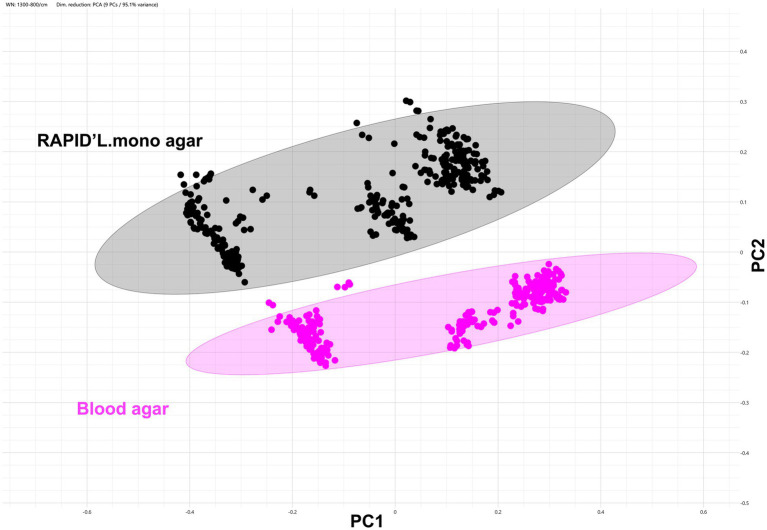
Growth media strongly influences the FTIR spectral profiles of *Listeria monocytogenes* strains. Data are shown as a 2D scatter plot of the FTIR spectra from 32 representative *Listeria monocytogenes* strains grown on blood agar (magenta) and Rapid’Lmono agar (black) at 37 °C for 24 h. Each symbol (•) corresponding to a technical replicate derived from at least two independent biological replicates per strain. The analysis was based on the 1300–800 cm^−1^ spectral window. Dimensionality reduction was performed using PCA with 9 PCs, capturing 95.1% of the variance. The plot displays the first two PC axes. The ellipsoids indicate the 95% CI (confidence interval).

Growth conditions have a significant impact on cellular composition by influencing gene expression and metabolic activity, which in turn can affect FTIR-based strain typing. To evaluate this impact, we assessed FTIR’s ability to distinguish *L. monocytogenes* strains under various growth conditions, including different media types, incubation temperatures, and durations. Across all conditions, FTIR successfully differentiated isolates, clustering them by strain and serogroup with high accuracy (Supplementary Table S4). The strongest clustering coherence was observed when isolates were grown on the same medium. When attempting to cluster isolates grown on different media under the same incubation conditions (temperature, duration), we found that the type of growth media had a dominant effect on cellular composition, significantly influencing FTIR spectral profiles and clustering. For example, spectra from the same strains grown on Blood agar and RAPID’L.mono agar clustered separately, primarily based on the media type (Figure 4). Instead of clustering by strain, the spectra aligned more closely with other strains grown on the same medium, highlighting the media’s strong influence on FTIR spectral profiles. Interestingly, despite the media influence, strains from the same serogroup grown on the same medium still clustered together (Supplementary Figure S6).

**Figure 5:**
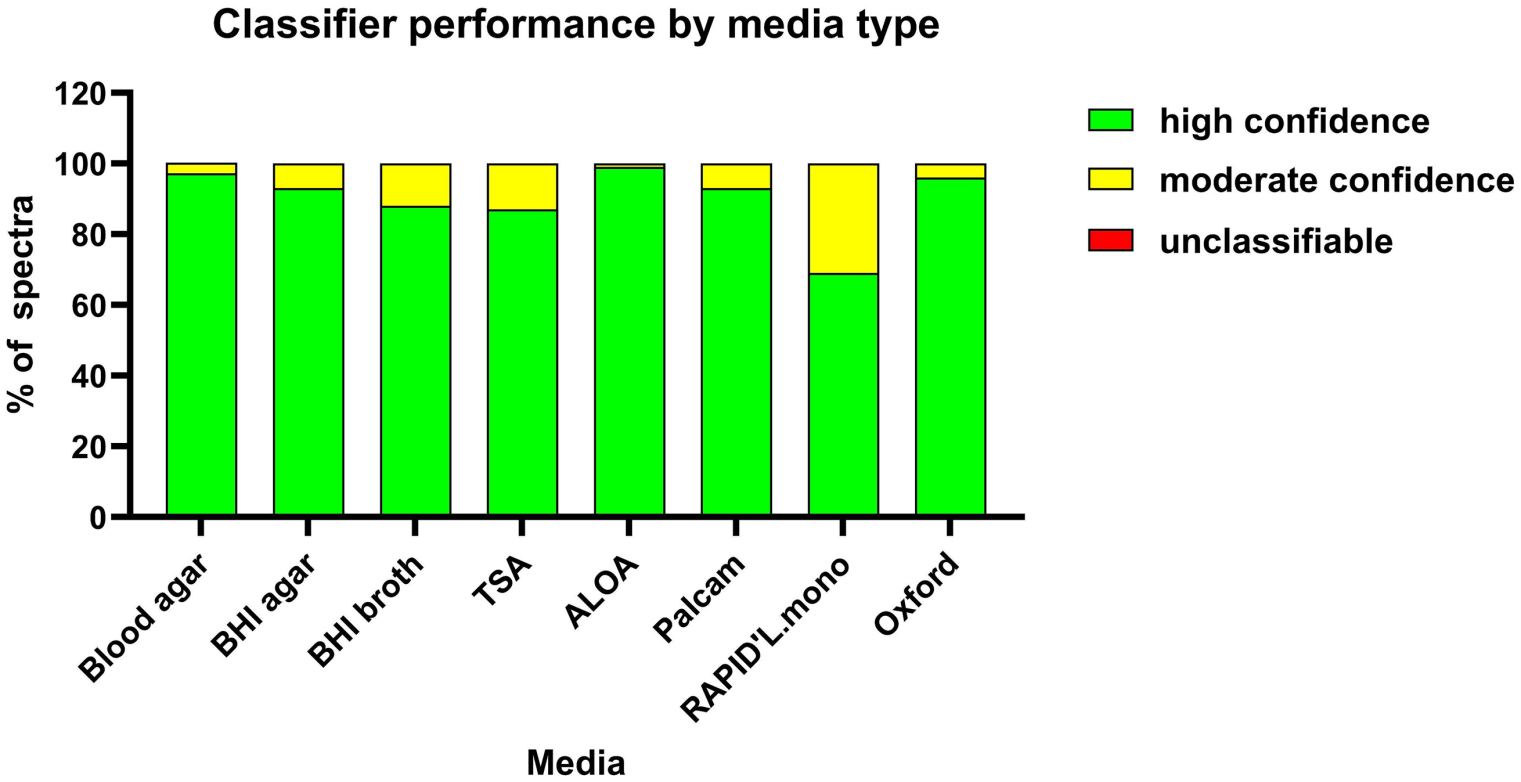
Classifier performance across different media types. *Listeria monocytogenes* strains (*n* = 32) were cultured on eight different media for 24 h at 37 °C and analysed by FTIR spectroscopy. The bar graph shows the percentage of spectra for each media type, classified as green, yellow, or red, relative to the total number of spectra per media, from a minimum of two biological replicates. Classification scores are color-coded: green (<1.0) indicates spectra within the classifiers training set boundaries and considered reliable, yellow (1.0–2.0) represents spectra near the boundaries and considered moderately reliable, and red (>2.0) indicates spectra outside the training set boundaries, deemed unreliable or unclassifiable.

Spectra from the same 32 strains grown on eight different media types were correctly classified, aligning fully with serological and WGS serotyping results (ARI = 1.00). None of the spectra from colonies across all media types received a red classification score (unclassified). However, classification confidence varied across media types, with the ranking, ALOA > Blood agar > Oxford > BHI > Palcam > BHI broth > TSA > RAPID’L.mono (Figure 5). Isolates grown on RAPID’L.mono agar exhibited the highest proportion of yellow classification scores (30.3%, 102/337), indicating results near the boundaries of the classifier training set that should be interpreted with caution (Figure 5; Supplementary Table S4). In contrast, ALOA performed the best, with only 0.8% of spectra having yellow scores. Colonies from ALOA and Blood agar plates (3% yellow) were the easiest to harvest and resuspend in ethanol. On the other hand, colonies from BHI and TSA agar were sticky, difficult to harvest and resuspend, possibly due to their higher hydrophilicity compared to colonies grown on other media types. Although spectra from colonies grown on Oxford agar showed high classification confidence (95.7% green scores), these colonies were more challenging to harvest from the agar plates.

#### 3.4.1 Impact of growth medium type

**Figure 6:**
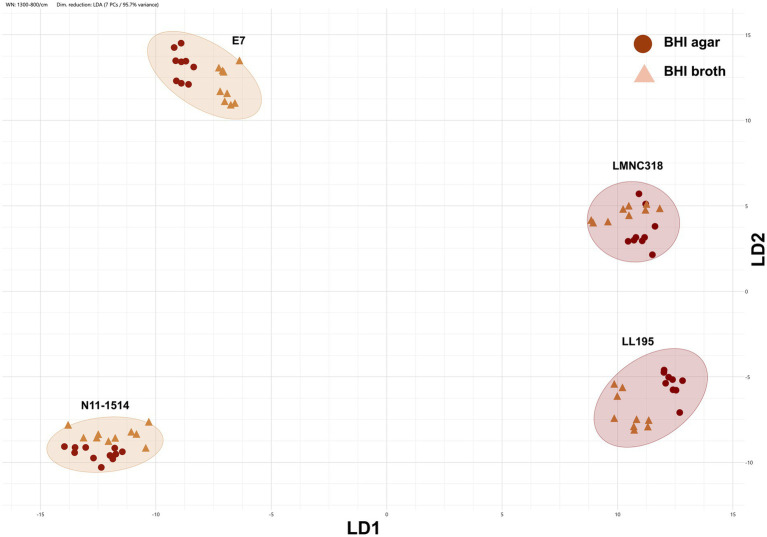
Impact of culture media on FTIR-based clustering. 2D scatter plot derived from FTIR spectra of *Listeria monocytogenes* strains E7, N11-1514, LL195, and LMNC318 grown on BHI agar or in BHI broth for 24 h at 37 °C. Technical replicates (symbols) from at least three biological replicates are shown. Points are color- and shape-coded by growth medium (BHI agar: maroon circles; BHI broth: light brown triangles). The analysis was based on the 1,300–800 cm^−1^ spectral window. Dimensionality reduction was performed using LDA with 7 principal components (PCs), capturing 95.5% of the variance.

To further explore the impact of solid versus liquid media, a subset of strains was cultured on BHI agar and in BHI broth under identical incubation conditions in terms of temperature and duration. FTIR spectra from agar and broth preparations of the same strain clustered closely, though with some separation, and remained distinct from those of other isolates, regardless of the growth medium (Figure 6). Liquid cultures showed potential for more standardized sample preparation due to precise culture volume control, suggesting a promising approach for refining FTIR-based methodologies. However, culturing isolates in BHI broth at 37 °C did not improve serotype-level clustering compared to BHI agar under the same conditions and, in fact, resulted in reduced classification scores for some strains (Figure 5). This outcome is expected, as the classifier was originally trained using spectra from isolates grown on BHI agar rather than broth.

#### 3.4.2 Impact of temperature and incubation duration on FTIR-based clustering

**Figure 7:**
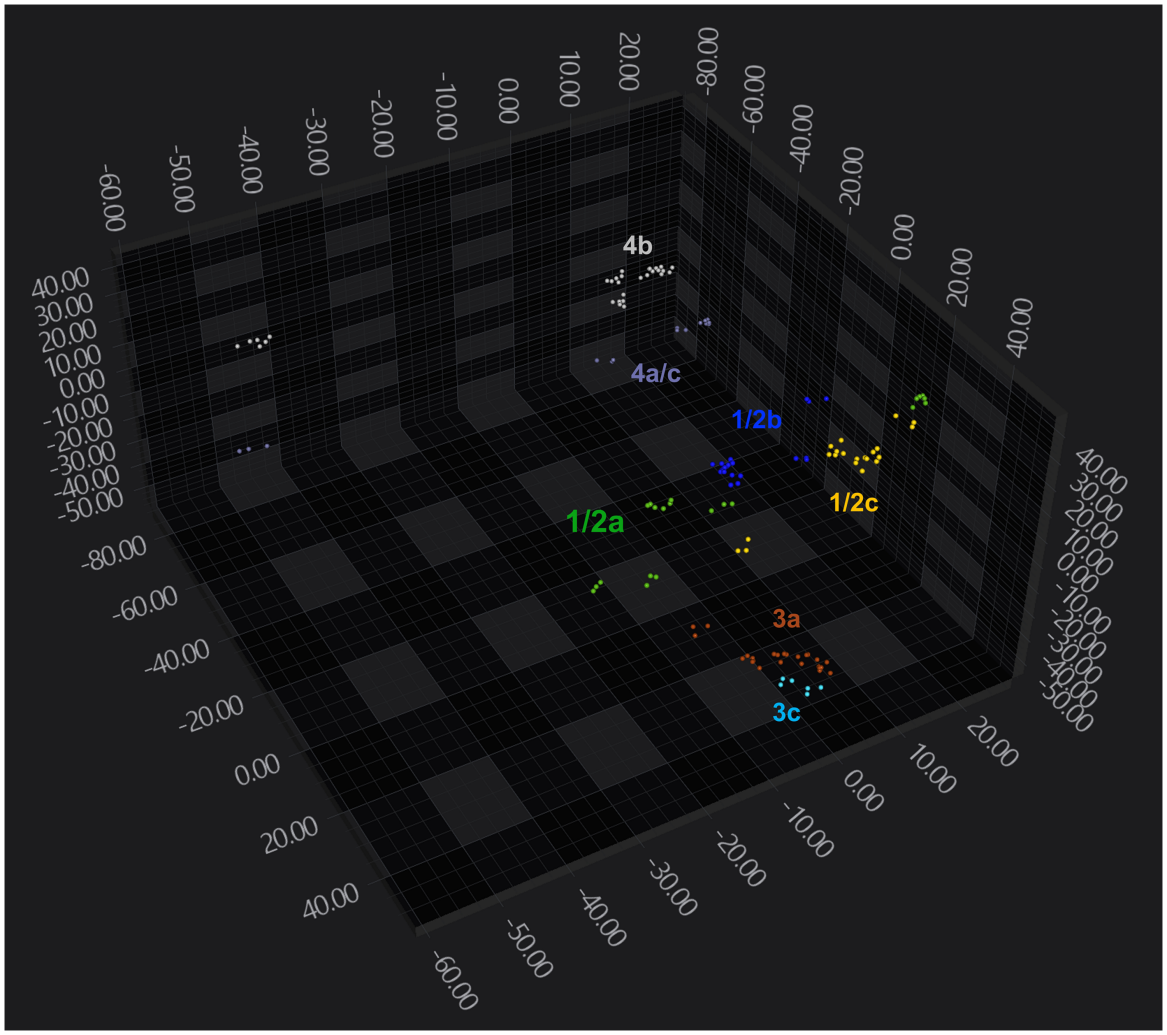
Clustering of *Listeria monocytogenes* strains based on serotype. 3D scatter plot showing the distribution of FTIR spectra of *Listeria monocytogenes* strains within the spectral space. The plot displays the first three LD axes, with spectra color-coded by serotype (green: 1/2a; blue: 1/2b; yellow: 1/2c; brown: 3a; cyan: 3c; purple: 4a/c; grey: 4b). Each symbol (•) corresponding to a technical replicate from at least two independent biological repeats. All cultures were grown on Rapid’Lmono agar at 25 °C for 24 h. The analysis was based on the 1,300–800 cm^−1^ spectral window. Dimensionality reduction was performed using LDA with 8 PCs, capturing 95.6% of the variance.

To investigate the impact of temperature on FTIR-based clustering, a subset of strains was cultivated on different media at 25 °C for 24 h, aiming to enhance differentiation of serotypes within the same serogroup. FTIR spectroscopy successfully clustered isolates grown at 25 °C by their strain and serogroup. The results showed improved differentiation of serotypes within the 1/2 and 3 serogroups, although some overlap persisted within serogroup 1/2, between serotype 1/2a and 1/2c (Figure 7). This enhancement was also observed when analysing the data using alternative spectral windows (Supplementary Figure S7). However, these alternative windows resulted in increased overlap among serogroup 4 serotypes, and the differentiation of serotypes within serogroup 1/2 and 3 was less pronounced compared to the default spectral window (1,300–800 cm^−1^). Using the manufacturer’s *Lmono* serogroup classifier, all spectra from cultures grown at 25 °C were assigned the correct serogroups, though some strains exhibited lower classification confidence scores (Supplementary Table S5). This was expected, as the classifier was originally trained on strains incubated at 37 °C. Overall, our results suggest that while the effect of temperature on FTIR spectra profiles is minimal, it is significant enough to induce observable differences in clustering patterns.

Similarly, incubation time had a small yet observable impact on clustering outcomes. Strains grown for 20, 24, or 48 h on the same media displayed consistent clustering patterns (data not shown). However, spectra from some strains (e.g., N1546, EGDe, N2306, LL196, and LMNC318) grown on Blood agar, ALOA, and BHI agar after 48 h of incubation showed lower confidence scores for serogroup prediction (Supplementary Table S6). These results suggest that extended incubation periods may alter cellular composition, which could, in turn, affect FTIR spectra. For strains incubated for 20 h, several failed to meet the quality standards due to low absorbance (<0.4), which was likely caused by insufficient colony growth during the shorter incubation period.

#### 3.4.3 Analysis of spectral regions affected by growth conditions

**Figure 8:**
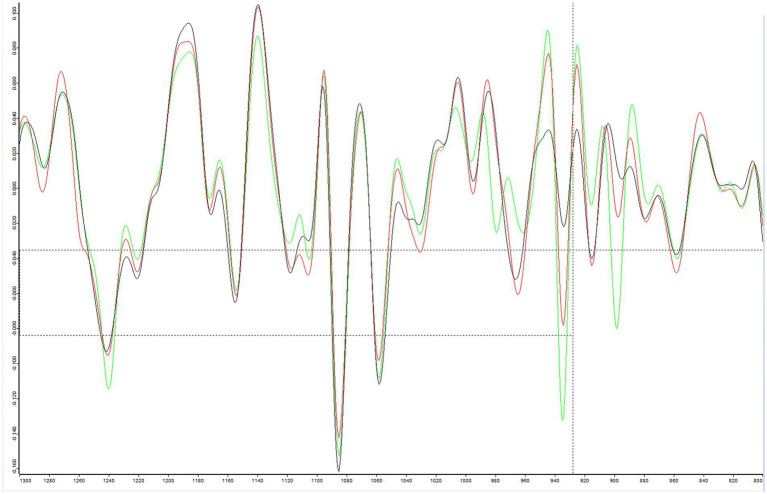
Representative vector-normalized second-derivative spectra of *Listeria monocytogenes* strain LL195, generated using OPUS software, are presented for visualization. The strain was cultured at 37 °C for 24 h on ALOA (green), blood agar (red), and RAPID’L.mono agar (dark grey). The spectral range displayed is 1,300–800 cm^−1^.

We next investigated the spectral regions influenced by different growth conditions. Our analysis revealed that several spectral regions were significantly affected by both growth media and temperature, including the lipid (3,000–2,800 cm^−1^), amide (1,800–1,500 cm^−1^), and polysaccharide (1,300–800 cm^−1^) regions (Supplementary Figures S8–S10). Comparative spectral analysis of the same strains incubated for 24 h on various media, including ALOA, Blood agar, and RAPID’L.mono agar, showed notable differences in the polysaccharide region (1,300–800 cm^−1^), which is commonly used for strain classification (Figure 8). The most pronounced spectral variations were observed in the 1,130–1,100 cm^−1^ and 1,000–860 cm^−1^ regions.

### 3.5 Potential of FTIR as a real-time tool for strain cluster analysis in outbreak and persistence investigations

**Figure 9:**
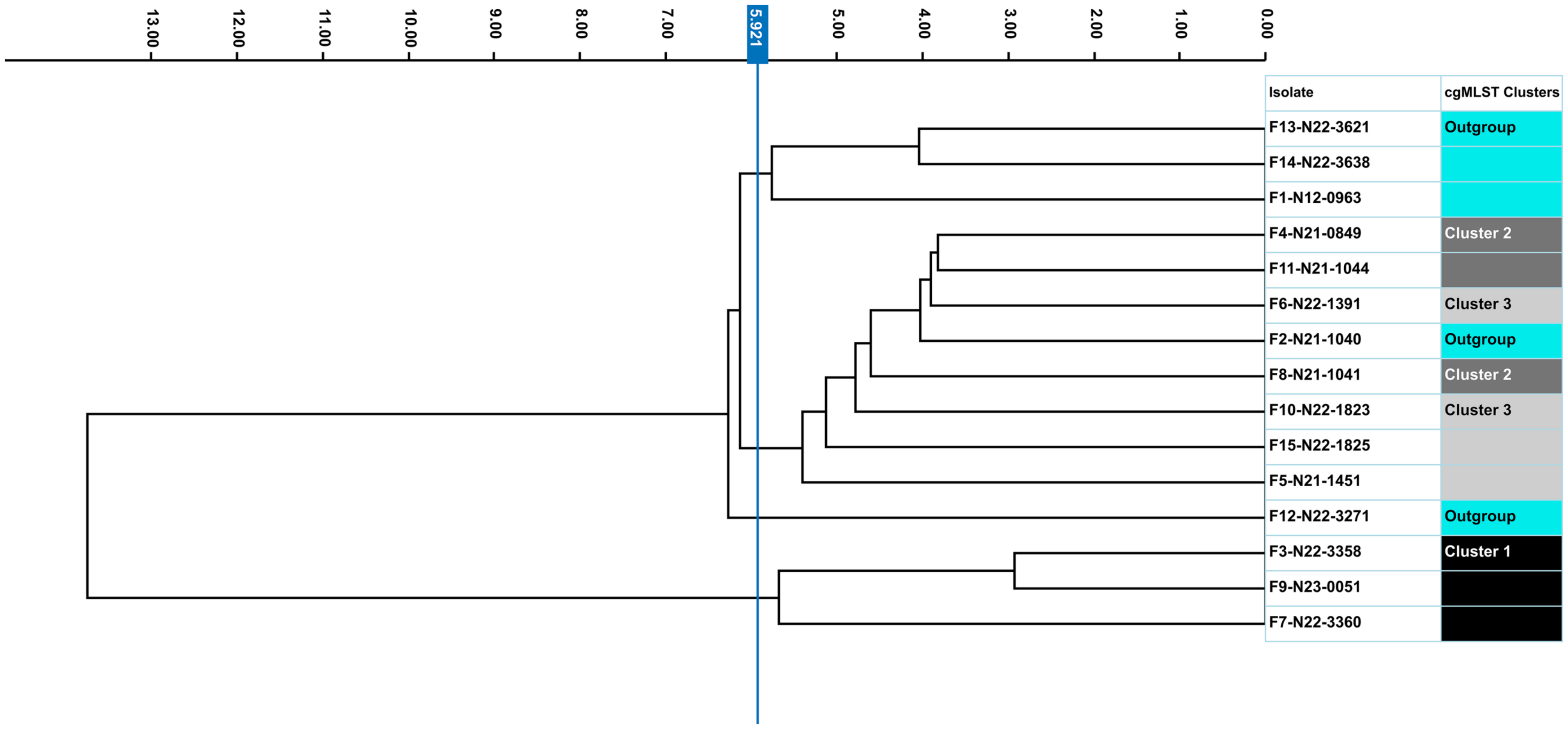
Evaluation of FTIR as a real-time cluster analysis tool. Dendrogram showing the clustering of FTIR spectra of *Listeria monocytogenes* CC9 strains from three cgMLST clusters and outgroup strains. Each strain is color-coded based on its cgMLST cluster. Rows represent average spectra, which were derived from two independent experiments each with three technical replicates using cultures grown on Rapid’Lmono agar at 37 °C for 24 h. The vertical blue line indicates the analysis clustering cut-off value (5.921). The analysis was based on the 1,300–800 cm^−1^ spectral window. Dimensionality reduction was performed using LDA (14 LDs of 15 PCs), capturing 95.1% of the variance.

**Figure 10:**
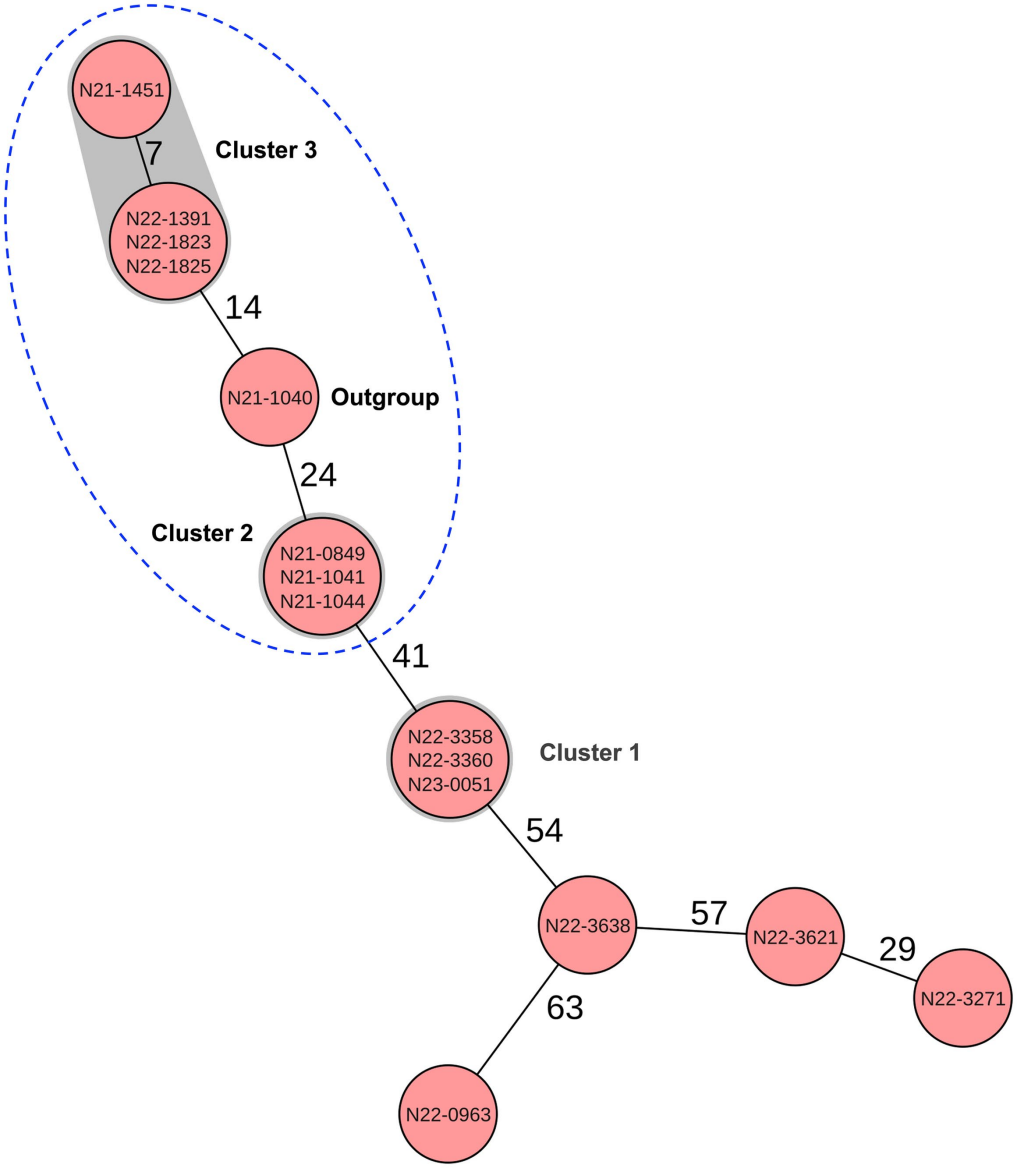
Minimum-spanning tree depicting the phylogenetic relationships among the 15 isolates analysed in the blinded clustering analysis study, based on cgMLST allelic profiles. Each circle represents a unique allelic profile derived from 1,701 cgMLST target genes. Numbers on connecting lines indicate the number of allelic differences between isolates. Clonal strains, defined as those with ≤10 allelic differences, are represented within the same circle or linked by gray shading, and are annotated by outbreak. The dotted blue ellipse highlights strains from cgMLST based clusters 2 and 3, along with the outgroup strain, which clustered together by FTIR.

To assess the potential of FTIR for real-time cluster identification in outbreak or persistence investigations, we conducted a blinded analysis in which no genomic clustering information was available during FTIR-based interpretation. Cluster assignments and interpretations were finalized prior to unblinding, allowing FTIR performance to be evaluated independently of cgMLST results. FTIR showed a strong ability to group the genetically related *L. monocytogenes* CC9 isolates originating from outbreak or persistence scenarios, although some overlap with outgroup isolates was observed (Figure 9; Supplementary Figure S11). Isolates belonging to cgMLST cluster 1 (F3: N22-3358, F7: N22-3360, and F9: N23-0051) formed a clearly distinct cluster, separated from both other cgMLST clusters and outgroup controls. In contrast, isolates from cgMLST clusters 2 and 3 exhibited partial overlap with each other and with a genetically closer outgroup strain (F2: N21-1041), despite over 10 cgMLST allele genetic differences among these strains and controls (Figure 10). Although FTIR lacked the high resolution of WGS, it effectively excluded most unrelated isolates, thereby narrowing the pool of candidates requiring downstream sequencing. This selective clustering highlights FTIR’s utility as a rapid, preliminary screening tool for strain cluster identification, enabling targeted WGS analysis by prioritizing potentially related isolates. Such early-stage grouping is particularly valuable in outbreak and persistence investigations, where fast decision-making can substantially reduce analytical burden and turnaround time.

### 3.6 Comparison of FTIR with other typing methods

We compared the typing efficiency of FTIR with other methods, including serological serotyping, MLST, PCR serogrouping, and WGS, to assess the discriminatory power of each technique. SID was used to quantify the diversity detected by each method. Among the methods tested, MLST exhibited the highest diversity-detecting ability, with a SID of 0.938 and 26 partitions. This was followed by serological serotyping and WGS serotyping (SID = 0.744 and 0.731, respectively, both with 9 partitions), and then PCR serogrouping (SID = 0.704, 5 partitions). FTIR typing had the lowest diversity index (SID = 0.574, 3 partitions), as it could only predict the serogroup, while other methods (e.g., serotyping) could determine the serotype. Despite its lower resolution, FTIR-based serogroup assignment showed strong concordance with serological serotyping, with an ARI and AWC of 1.000. Agreement between FTIR and WGS serogroups was also high, with an ARI of 0.948 and AWC of 0.987. The only discordant strains were N843_10 and N843_15, which were assigned to serogroup 3 by both FTIR and serological serotyping but classified as serogroup 1/2 by WGS.

## 4 Discussion

This study demonstrates that FTIR spectroscopy is a robust and reproducible phenotypic approach for rapid *L. monocytogenes* strain typing. Across a diverse collection of well-characterized strains, FTIR showed high reproducibility and strong discriminatory power at the strain and serogroup levels, aligning with traditional serotyping methods and WGS-based assignments. With a turnaround time of approximately 3 hours per 30 isolates, these findings support the suitability of FTIR for high-throughput applications where rapid preliminary classification and exclusion of unrelated isolates are required, with WGS serving as a confirmatory high-resolution method.

As expected for a phenotypic technique, FTIR showed limited resolution for fine-scale differentiation based on MLST or for distinguishing closely related serotypes within the same serogroup, particularly among serotypes 1/2a, 1/2b, and 1/2c. This limitation likely reflects spectral overlap in polysaccharide-associated regions that dominate FTIR-based classification of *L. monocytogenes*. Similar constraints have been reported for other phenotypic typing methods, highlighting fundamental differences between biochemical fingerprinting and genome-based approaches. Consequently, FTIR should not be viewed as a replacement for WGS, but rather as a complementary tool designed to address different operational needs.

Some *L. monocytogenes* MLST CCs encompass multiple serotypes, complicating FTIR-based differentiation at this level. For example, CC9 includes isolates from serotypes 1/2a, 1/2c, and 3c (Muchaamba et al., 2021), which likely contributes to the observed clustering challenges. In contrast, FTIR studies on other species, such as *Lactiplantibacillus plantarum* and *Bifidobacterium longum* subsp. *longum*, demonstrated higher FTIR resolution than MLST and performance comparable to WGS or PFGE (Deidda et al., 2021; Li et al., 2022). In *B. animalis* subsp. *lactis*, FTIR provided superior differentiation of strains compared to MLST and PFGE. Thus FTIR’s ability to differentiate isolates by MLST might be species-specific. However, these studies analysed smaller strains sets (10–20) with fewer MLST CCs, whereas our study included a larger, more diverse strain collection, likely explaining the lower MLST level resolution.

The ANN-based serogroup classifier in the IR Biotyper® system performed consistently across selective and non-selective culture media, including ISO-relevant chromogenic agars, with high agreement to serological serotyping (ARI and AWC = 1.00) and WGS-based assignments (ARI = 0.948 and AWC = 0.987). Importantly, this study extends previous work by demonstrating that reliable serogroup assignment can be achieved without additional subculturing on non-selective media, thereby improving compatibility with routine food testing workflows. While classification confidence varied between media types, no systematic misclassification was observed, highlighting the robustness of the approach when growth conditions are standardized. These findings align with those of Oberreuter et al. (2023), who first evaluated the IR Biotyper® using the manufacturer-provided ANN classifier for *L. monocytogenes*, albeit with Blood agar only.

Rebuffo et al. (2006) and Rebuffo-Scheer et al. (2007) used ANN-based analysis of spectral regions “3,100 to 2,800 cm^−1^, 1,800 to 1,500 cm^−1^, and 1,200 to 700 cm^−1^” and “1,200 to 900 cm^−1^ and 1,800 to 1,400 cm^−1^,” respectively, to differentiate *Listeria* species, and *L. monocytogenes* serogroups and serotypes. Our study expanded these approaches, incorporating the 1,300 to 800 cm^−1^ and 1,200 to 900 cm^−1^ windows for strain and serogroup differentiation. While serogroup agreement was comparable (98.8% vs. 100% with serological serotyping) using all four spectral windows, serotype resolution, which reached 91.6% in the other studies (Rebuffo-Scheer et al., 2007), was not assessed. According to the manufacturer, the current IR Biotyper® classifier is not yet effective for serotype assignment. Differences in classifier architecture, including training cycles and principal component selection, likely explain this resolution discrepancy. In the study by Rebuffo-Scheer et al. (2007) they used NeuroDeveloper™ software (Synthon GmbH), which employed a four-level hierarchical ANN, while our study classifier (IR Biotyper) relied on 30 PCs and 200 training cycles, focusing on the 1,300 to 800 cm^−1^ window, preselected for serogroup differentiation. Therefore, refining spectral windows and classifier design may improve discrimination, particularly for closely clustered serotypes or MLST-specific profiles. Additionally, such refinements could enable FTIR-based analysis to provide risk assessment insights. For example, FTIR was able to cluster 89 *L. monocytogenes* isolates into two groups, which corresponded with their susceptibilities to sakacin P, a class IIa bacteriocin (Oust et al., 2006).

Growth conditions were identified as a major determinant of spectral variability. Media type affected clustering behavior more than incubation duration, with spectra from the same strain grown on different media often clustering separately. This emphasizes the importance of harmonized cultivation protocols and explains part of the inter-study variability reported in the literature (Hong et al., 2022; Hu et al., 2023). For example, Hu et al. (2023) demonstrated superior discriminatory power for *P. aeruginosa* grown on Mueller-Hinton agar compared to Blood agar, used in the study by Zendri et al. (2024). In our study, FTIR classification accuracy was maintained across all eight tested media types. However, classification confidence varied, with ALOA and Blood agar performing best, providing the most consistent, high-quality spectra. In contrast, RAPID’L.mono and BHI broth yielded lower classifications confidence scores, likely due to media-driven changes in cellular metabolic states and molecular composition.

Although liquid cultures require longer processing times due to multiple washing steps, they offer greater control over sample biomass and uniformity, making them a promising avenue for refining FTIR protocols. In our study, FTIR spectra of the same strain cultivated in BHI broth or BHI agar clustered closely, indicating retention of strain-specific spectral patterns, though some medium-induced separation was observed. This further highlights the need for consistent standardized cultivation protocols to minimize growth condition-induced spectral variability. Contrary to previous findings in other species, such as *L. plantarum* (Deidda et al., 2021), culturing *L. monocytogenes* in BHI broth at 37 °C did not improve discriminatory power at the serotype-level. In fact, classification scores decreased for some broth-grown strains compared to agar-grown counterparts, likely due to a mismatch between the sample preparation method and the ANN classifier, which was trained on spectra from agar-grown isolates. Therefore, aligning growth conditions with classifier training data is critical for optimal FTIR performance.

In a study by Davis and Mauer (2011), liquid cultures were chosen over solid media for FTIR analysis of *L. monocytogenes*. They hypothesized that faster bacterial growth in liquid media results in more uniform physiological states, whereas on solid media, colonies may exhibit heterogeneous physiological characteristics due to variations in colony size and age. This heterogenicity can negatively impact FTIR reproducibility (Choo-Smith et al., 2001; Sandt et al., 2006). Adding to the challenges of using agar cultures, previous studies (Janbu et al., 2008), including our own, have reported difficulties in harvesting or resuspending colony material from some types of agar media in ethanol. This is likely due to the hydrophilic surface properties of *L. monocytogenes* grown on these media. Additionally, the risk of media carryover is higher with agar plates than with broth, potentially interfering with the spectral profile and reducing classification confidence. To address these challenges, future research should pursue the optimization of liquid culture conditions to minimize spectral variation caused by colony heterogeneity, media carryover, and harvesting difficulties, ultimately improving FTIR’s reproducibility and discriminatory performance for *L. monocytogenes* strain typing.

While incubation duration did not significantly affect clustering patterns, it did influence classification confidence. Prolonged incubation (48 h) led to reduced confidence. This was expected, as the classifier was optimized for strains grown at 37 °C for 24 h. Conversely, a 20-h incubation maintained clustering accuracy for most strains, but insufficient colony growth resulted in suboptimal spectral quality for some. Although shortening the incubation period could accelerate results, it risks compromising spectra quality. Lowering the incubation temperature to 25 °C, where *L. monocytogenes* strongly expresses flagella, improved clustering, particularly for serotype differentiation within the 1/2 and 3 serogroups. This suggests that targeted growth conditions optimization may enhance FTIR’s resolution. However, it is important to note that these findings were based on an investigation of only 32 strains, representing 7 of the 14 serotypes of *L. monocytogenes*. Therefore, further studies with a broader range of strains and serotypes are needed to confirm the generalizability of these results.

In a blinded, outbreak-like cluster analysis, FTIR effectively grouped genetically related CC9 isolates and excluded most unrelated strains, despite limited resolution among very closely related cgMLST clusters. While FTIR could not fully resolve all genomic relationships, its ability to rapidly narrow the pool of candidate isolates demonstrates clear practical value. In outbreak and persistence investigations, this form of early-stage phenotypic triage can substantially reduce the number of isolates requiring immediate WGS (Jacobs et al., 2025), thereby improving turnaround times and optimizing sequencing resources. Establishing a cutoff based on cgMLST allelic differences would also aid in defining FTIR’s limit of detection in outbreak settings, ensuring that the tool is applied appropriately for various levels of relatedness. The most recent version of the IR Biotyper® software incorporate additional machine-learning approaches, including k-nearest neighbors (kNN), which may offer enhanced flexibility for FTIR-based classification. Preliminary exploratory application of kNN produced clustering trends broadly consistent with the outbreak cluster analysis, however, in the absence of validated performance benchmarks and standardized cut-off criteria for defining relatedness or clonality in *L. monocytogenes*, these results were not included. Systematic validation and standardization of kNN-based FTIR analyses, as well as other machine-learning models, should therefore be addressed in future work.

Compared to PCR serogrouping, FTIR offers a simpler workflow. Although its serogroup resolution is currently lower than PCR when using the existing IR Biotyper® serogroup classifier, FTIR demonstrates superior discriminatory power by enabling strain-level differentiation, which is not possible with PCR serogrouping. FTIR has also shown potential for differentiating serotypes, as demonstrated by our findings with cultures grown at 25 °C and in the work of Rebuffo-Scheer et al. (2007), who applied a four-level hierarchical ANN for serotype classification. Overall, FTIR’s ability to differentiate both strains and, in some cases, serotypes makes it a potentially more powerful tool for strain typing than PCR which only identifies serogroups (Doumith et al., 2004; Vitullo et al., 2013).

A key limitation of FTIR and similar phenotypic methods is susceptibility to phenotypic changes driven by small genetic mutations. In our study, strains N843_10 and N843_15 showed discordance in serotype classification, both were assigned to serogroup 3 by FTIR- and serological serotyping but classified as serogroup 1/2 by WGS. These strains, isolated from the same prosthetic hip joints 5 years apart, may have diverged genetically due to adaptation in the prosthetic joint environment. Notably, N843_15 underwent multiple genetic changes, likely driven by random mutations or selective pressure (Muchaamba et al., 2020). Such mutations can cause phenotypic changes that alter serotype classification, even in clonally related strains. This is consistent with other studies where clonally identical strains exhibited phenotypic differences, such as hemolysis, due to single SNPs (Charlier et al., 2012). This discordance highlights the limitations of phenotypic serotyping methods, as subtle genetic variations in key genes can significantly impact results. In contrast, WGS can detect and quantify these differences, making it more reliable for fine-scale strain differentiation, particularly when phenotypic changes arise from small genetic mutations. Furthermore, as with most typing methods, FTIR analysis depends on pure cultures for accurate strain identification. The spectral profiles of mixed cultures differ significantly from those of pure cultures, which can affect the accuracy of strain typing and differentiation (Nyarko and Donnelly, 2015).

Differentiation of serotype 4b from other serogroup 4 strains appears feasible, provided that enough non-serotype 4b isolates are included in the reference database. However, the current database contains only a limited number of non-serotype 4b serogroup 4 strains, which may affect classification robustness. A key limitation of our study is the underrepresentation, or complete absence, of some serotypes, including 3b, 3c, 4a, 4c, 4d, 4e, 4h, and 7, within our strain collection. This gap may hinder the reliability of FTIR-based classification, particularly for these serotypes. To address this, future studies should expand the strain database by incorporating a broader range of isolates from environmental, clinical, and food sources. Collaborative efforts with reference laboratories and strain repositories could facilitate access to rare serotypes, ultimately improving FTIR spectroscopy discriminatory power and reliability for *L. monocytogenes* serotype differentiation. Further enhancements could be achieved by refining the classifier with secondary models for serotype differentiation, which may improve both the accuracy and overall utility of FTIR systems for *L. monocytogenes* strain typing.

Overall, our findings confirm FTIR as a fast, accessible, and scalable first-line screening tool that complements WGS rather than competes with it. Although it may not resolve fine-scale distinctions like WGS, its primary strength lies in reproducibility, speed, and dereplication capacity, enabling efficient prioritization of isolates for downstream genomic analysis. However, the strong influence of growth conditions on spectral profiles highlights the need to apply optimized FTIR protocols to improve reproducibility and discriminatory power, particularly at the serotype level. Continued expansion of reference databases, optimization of cultivation protocols, and validation of emerging classification algorithms (e.g., kNN) are expected to further enhance the operational value of FTIR-based typing in food safety surveillance and outbreak response.

## Data Availability

The datasets presented in this study can be found in online repositories. The names of the repository/repositories and accession number(s) can be found in the article/Supplementary material.

## Footnotes

1
http://www.comparingpartitions.info/

## References

[ref1] Al-Fraihat E. Barker K. R. Tadros M. (2025). The IR Biotyper as a tool for typing organisms of significance for hospital epidemiology- a subject review. Diagn. Microbiol. Infect. Dis. 111:116676. doi: 10.1016/j.diagmicrobio.2024.116676, 39756141

[ref2] Buchanan R. L. Gorris L. G. Hayman M. M. Jackson T. C. Whiting R. C. (2017). A review of *Listeria monocytogenes*: an update on outbreaks, virulence, dose-response, ecology, and risk assessments. Food Control 75, 1–13. doi: 10.1016/j.foodcont.2016.12.016

[ref3] Carriço J. A. Silva-Costa C. Melo-Cristino J. Pinto F. R. de Lencastre H. Almeida J. S. . (2006). Illustration of a common framework for relating multiple typing methods by application to macrolide-resistant *Streptococcus pyogenes*. J. Clin. Microbiol. 44, 2524–2532. doi: 10.1128/jcm.02536-05, 16825375 PMC1489512

[ref4] Charlier C. Leclercq A. Cazenave B. Desplaces N. Travier L. Cantinelli T. . (2012). *Listeria monocytogenes*-associated joint and bone infections: a study of 43 consecutive cases. Clin. Infect. Dis. 54, 240–248. doi: 10.1093/cid/cir803, 22100574

[ref5] Choo-Smith L. P. Maquelin K. van Vreeswijk T. Bruining H. A. Puppels G. J. Ngo Thi N. A. . (2001). Investigating microbial (micro)colony heterogeneity by vibrational spectroscopy. Appl. Environ. Microbiol. 67, 1461–1469. doi: 10.1128/AEM.67.4.1461-1469.2001, 11282591 PMC92755

[ref6] Cordovana M. Mauder N. Join-Lambert O. Gravey F. LeHello S. Auzou M. . (2022). Machine learning-based typing of *Salmonella enterica* O-serogroups by the Fourier-transform infrared (FTIR) spectroscopy-based IR Biotyper system. J. Microbiol. Methods 201:106564. doi: 10.1016/j.mimet.2022.106564, 36084763

[ref7] Davis R. Mauer L. J. (2011). Subtyping of *Listeria monocytogenes* at the haplotype level by Fourier transform infrared (FT-IR) spectroscopy and multivariate statistical analysis. Int. J. Food Microbiol. 150, 140–149. doi: 10.1016/j.ijfoodmicro.2011.07.024, 21864929

[ref8] Deidda F. Bozzi Cionci N. Cordovana M. Campedelli I. Fracchetti F. Di Gioia D. . (2021). Bifidobacteria strain typing by Fourier transform infrared spectroscopy. Front. Microbiol. 12:692975. doi: 10.3389/fmicb.2021.692975, 34589064 PMC8473902

[ref9] Doumith M. Buchrieser C. Glaser P. Jacquet C. Martin P. (2004). Differentiation of the major *Listeria monocytogenes* serovars by multiplex PCR. J. Clin. Microbiol. 42, 3819–3822. doi: 10.1128/JCM.42.8.3819-3822.2004, 15297538 PMC497638

[ref10] Duchenne-Moutien R. A. Neetoo H. (2021). Climate change and emerging food safety issues: a review. J. Food Prot. 84, 1884–1897. doi: 10.4315/JFP-21-141, 34185849

[ref11] Duma M. N. Ciupescu L. M. Dan S. D. Crisan-Reget O. L. Tabaran A. (2024). Virulence and antimicrobial resistance of *Listeria monocytogenes* isolated from ready-to-eat food products in Romania. Microorganisms 12:954. doi: 10.3390/microorganisms12050954, 38792784 PMC11123701

[ref12] Duze S. T. Marimani M. Patel M. (2021). Tolerance of *Listeria monocytogenes* to biocides used in food processing environments. Food Microbiol. 97:103758. doi: 10.1016/j.fm.2021.103758, 33653529

[ref13] Fagerlund A. Langsrud S. Møretrø T. (2021). Microbial diversity and ecology of biofilms in food industry environments associated with *Listeria monocytogenes* persistence. Curr. Opin. Food Sci. 37, 171–178. doi: 10.1016/j.cofs.2020.10.015

[ref14] Finn L. Onyeaka H. O'Neill S. (2023). *Listeria monocytogenes* biofilms in food-associated environments: a persistent enigma. Foods 12:3339. doi: 10.3390/foods12183339, 37761048 PMC10529182

[ref15] Gant M. S. Chamot-Rooke J. (2024). Present and future perspectives on mass spectrometry for clinical microbiology. Microbes Infect. 26:105296. doi: 10.1016/j.micinf.2024.105296, 38199266

[ref16] Gorski L. (2014). Serotype assignment by sero-agglutination, ELISA, and PCR. Methods Mol. Biol. 1157, 41–61. doi: 10.1007/978-1-4939-0703-8_4, 24792547

[ref17] Hong J. S. Kim D. Jeong S. H. (2022). Performance evaluation of the IR Biotyper® system for clinical microbiology: application for detection of *Staphylococcus aureus* sequence type 8 strains. Antibiotics 11:909. doi: 10.3390/antibiotics11070909, 35884163 PMC9311605

[ref18] Hu Y. Wang W. van Nguyen S. Macori G. Li F. Fanning S. (2024). Editorial: high-level antimicrobial resistance or hypervirulence in emerging and re-emerging "super-bug" foodborne pathogens: detection, mechanism, and dissemination from omics insights. Front. Microbiol. 15:1459601. doi: 10.3389/fmicb.2024.1459601, 39184029 PMC11344267

[ref19] Hu Y. Zhu K. Jin D. Shen W. Liu C. Zhou H. . (2023). Evaluation of IR Biotyper for carbapenem-resistant *Pseudomonas aeruginosa* typing and its application potential for the investigation of nosocomial infection. Front. Microbiol. 14:1068872. doi: 10.3389/fmicb.2023.1068872, 36846786 PMC9947493

[ref20] Hyden P. Pietzka A. Lennkh A. Murer A. Springer B. Blaschitz M. . (2016). Whole genome sequence-based serogrouping of *Listeria monocytogenes* isolates. J. Biotechnol. 235, 181–186. doi: 10.1016/j.jbiotec.2016.06.005, 27288594

[ref21] Jacobs B. Masquelier J. van Nieuwenhuysen T. Delbrassinne L. van Hoorde K. (2025). Employing fourier-transform infrared spectroscopy as dereplication strategy in foodborne outbreak investigation of cereulide-producing *Bacillus cereus*. Int. J. Food Microbiol. 447:111550. doi: 10.1016/j.ijfoodmicro.2025.111550 41338054

[ref22] Janbu A. O. Møretrø T. Bertrand D. Kohler A. (2008). FT-IR microspectroscopy: a promising method for the rapid identification of *Listeria* species. FEMS Microbiol. Lett. 278, 164–170. doi: 10.1111/j.1574-6968.2007.00995.x, 18053065

[ref23] Kim J. Ahn J. (2022). Emergence and spread of antibiotic-resistant foodborne pathogens from farm to table. Food Sci. Biotechnol. 31, 1481–1499. doi: 10.1007/s10068-022-01157-1, 36065433 PMC9435411

[ref24] Li X. Zhu L. Wang X. Li J. Tang B. (2022). Evaluation of IR Biotyper for Lactiplantibacillus plantarum typing and its application potential in probiotic preliminary screening. Front. Microbiol. 13:823120. doi: 10.3389/fmicb.2022.823120, 35401469 PMC8988154

[ref25] Lombardo D. Cordovana M. Deidda F. Pane M. Ambretti S. (2021). Application of Fourier transform infrared spectroscopy for real-time typing of *Acinetobacter Baumannii* outbreak in intensive care unit. Future Microbiol. 16, 1239–1250. doi: 10.2217/fmb-2020-0276, 34674538

[ref26] Lurie-Weinberger M. N. Temkin E. Kastel O. Bechor M. Bychenko-Banyas D. Efrati-Epchtien R. . (2025). Use of a national repository of Fourier-transform infrared spectroscopy spectra enables fast detection of silent outbreaks and prevention of spread of new antibiotic-resistant sequence types. Antimicrob. Resist. Infect. Control 14:34. doi: 10.1186/s13756-025-01546-1, 40259416 PMC12013074

[ref27] Maury M. M. Tsai Y.-H. Charlier C. Touchon M. Chenal-Francisque V. Leclercq A. . (2016). Uncovering *Listeria monocytogenes* hypervirulence by harnessing its biodiversity. Nat. Genet. 48, 308–313. doi: 10.1038/ng.3501, 26829754 PMC4768348

[ref28] Meijer G. W. Lähteenmäki L. Stadler R. H. Weiss J. (2021). Issues surrounding consumer trust and acceptance of existing and emerging food processing technologies. Crit. Rev. Food Sci. Nutr. 61, 97–115. doi: 10.1080/10408398.2020.1718597, 32003225

[ref29] Muchaamba F. Eshwar A. K. Ah U. von Stevens M. J. A. Tasara T. 2020 Evolution of *Listeria monocytogenes* during a persistent human prosthetic hip joint infection Front. Microbiol. 11:1726 doi: 10.3389/fmicb.2020.01726 32849369 PMC7399150

[ref30] Muchaamba F. Eshwar A. K. Stevens M. J. A. Stephan R. Tasara T. (2021). Different shades of *Listeria monocytogenes*: strain, serotype, and lineage-based variability in virulence and stress tolerance profiles. Front. Microbiol. 12:792162. doi: 10.3389/fmicb.2021.792162, 35058906 PMC8764371

[ref31] Muchaamba F. Stephan R. (2024). A comprehensive methodology for microbial strain typing using fourier-transform infrared spectroscopy. Methods Protoc. 7:48. doi: 10.3390/mps7030048, 38921827 PMC11207048

[ref32] Nyarko E. Donnelly C. (2015). Differentiation of different mixed Listeria strains and also acid-injured, heat-injured, and repaired cells of *Listeria monocytogenes* using fourier transform infrared spectroscopy. J. Food Prot. 78, 540–548. doi: 10.4315/0362-028X.JFP-14-160, 25719878

[ref33] Oberreuter H. Cordovana M. Dyk M. Rau J. (2025). Establishment and thorough external validation of an FTIR spectroscopy classifier for *Salmonella* serogroup differentiation. bioRxiv. doi: 10.48414/ASPECTS2025/16

[ref34] Oberreuter H. Dyk M. Rau J. (2023). Validated differentiation of *Listeria monocytogenes* serogroups by FTIR spectroscopy using an artificial neural network based classifier in an accredited official food control laboratory. Clin. Spectrosc. 5:100030. doi: 10.1016/j.clispe.2023.100030

[ref35] Orsi R. H. den Bakker H. C. Wiedmann M. (2011). *Listeria monocytogenes* lineages: genomics, evolution, ecology, and phenotypic characteristics. Int. J. Med. Microbiol. 301, 79–96. doi: 10.1016/j.ijmm.2010.05.002, 20708964

[ref36] Osek J. Lachtara B. Wieczorek K. (2022). *Listeria monocytogenes* - how this pathogen survives in food-production environments? Front. Microbiol. 13:866462. doi: 10.3389/fmicb.2022.866462, 35558128 PMC9087598

[ref37] Oust A. Møretrø T. Naterstad K. Sockalingum G. D. Adt I. Manfait M. . (2006). Fourier transform infrared and raman spectroscopy for characterization of *Listeria monocytogenes* strains. Appl. Environ. Microbiol. 72, 228–232. doi: 10.1128/AEM.72.1.228-232.2006, 16391047 PMC1352188

[ref38] Pascale M. R. Bisognin F. Mazzotta M. Girolamini L. Marino F. Dal Monte P. . (2022). Use of Fourier-transform infrared spectroscopy with IR Biotyper® system for *Legionella pneumophila* serogroups identification. Front. Microbiol. 13:866426. doi: 10.3389/fmicb.2022.866426, 35558114 PMC9090449

[ref39] Passaris I. Mauder N. Kostrzewa M. Burckhardt I. Zimmermann S. van Sorge N. M. . (2022). Validation of fourier transform infrared spectroscopy for serotyping of *Streptococcus pneumoniae*. J. Clin. Microbiol. 60:e0032522. doi: 10.1128/jcm.00325-22, 35699436 PMC9297836

[ref40] Pinto F. R. Melo-Cristino J. Ramirez M. (2008). A confidence interval for the Wallace coefficient of concordance and its application to microbial typing methods. PLoS One 3:e3696. doi: 10.1371/journal.pone.0003696, 19002246 PMC2577298

[ref41] Rakovitsky N. Frenk S. Kon H. Schwartz D. Temkin E. Solter E. . (2020). Fourier transform infrared spectroscopy is a new option for outbreak investigation: a retrospective analysis of an extended-spectrum-beta-lactamase-producing *Klebsiella pneumoniae* outbreak in a neonatal intensive care unit. J. Clin. Microbiol. 58:e00098. doi: 10.1128/jcm.00098-20, 32161093 PMC7180251

[ref42] Rebuffo C. A. Schmitt J. Wenning M. von Stetten F. Scherer S. (2006). Reliable and rapid identification of Listeria monocytogenes and Listeria species by artificial neural network-based Fourier transform infrared spectroscopy. Appl. Environ. Microbiol. 72, 994–1000. doi: 10.1128/AEM.72.2.994-1000.2006, 16461640 PMC1392910

[ref43] Rebuffo-Scheer C. A. Schmitt J. Scherer S. (2007). Differentiation of *Listeria monocytogenes* serovars by using artificial neural network analysis of Fourier-transformed infrared spectra. Appl. Environ. Microbiol. 73, 1036–1040. doi: 10.1128/AEM.02004-06, 17142376 PMC1800759

[ref44] Romanolo K. F. Gorski L. Wang S. Lauzon C. R. (2015). Rapid identification and classification of Listeria spp. and serotype assignment of *Listeria monocytogenes* using Fourier transform-infrared spectroscopy and artificial neural network analysis. PLoS One 10:e0143425. doi: 10.1371/journal.pone.0143425, 26600423 PMC4658148

[ref45] Ruppitsch W. (2016). Molecular typing of bacteria for epidemiological surveillance and outbreak investigation. Bodenkultur 67, 199–224. doi: 10.1515/boku-2016-0017

[ref46] Ruppitsch W. Pietzka A. Prior K. Bletz S. Fernandez H. L. Allerberger F. . (2015). Defining and evaluating a Core genome multilocus sequence typing scheme for whole-genome sequence-based typing of *Listeria monocytogenes*. J. Clin. Microbiol. 53, 2869–2876. doi: 10.1128/JCM.01193-15, 26135865 PMC4540939

[ref47] Sandt C. Madoulet C. Kohler A. Allouch P. De Champs C. Manfait M. . (2006). FT-IR microspectroscopy for early identification of some clinically relevant pathogens. J. Appl. Microbiol. 101, 785–797. doi: 10.1111/j.1365-2672.2006.02969.x, 16968290

[ref48] Severiano A. Carriço J. A. Robinson D. A. Ramirez M. Pinto F. R. (2011a). Evaluation of jackknife and bootstrap for defining confidence intervals for pairwise agreement measures. PLoS One 6:e19539. doi: 10.1371/journal.pone.0019539, 21611165 PMC3097183

[ref49] Severiano A. Pinto F. R. Ramirez M. Carriço J. A. (2011b). Adjusted Wallace coefficient as a measure of congruence between typing methods. J. Clin. Microbiol. 49, 3997–4000. doi: 10.1128/JCM.00624-11, 21918028 PMC3209087

[ref50] Stevens M. J. A. Stephan R. Horlbog J. A. Cernela N. Nüesch-Inderbinen M. (2024). Whole genome sequence-based characterization of *Campylobacter* isolated from broiler carcasses over a three-year period in a big poultry slaughterhouse reveals high genetic diversity and a recurring genomic lineage of *Campylobacter jejuni*. Infect. Genet. Evol. 119:105578. doi: 10.1016/j.meegid.2024.105578 38417639

[ref51] Urban L. Perlas A. Francino O. Martí-Carreras J. Muga B. A. Mwangi J. W. . (2023). Real-time genomics for one health. Mol. Syst. Biol. 19:e11686. doi: 10.15252/msb.202311686, 37325891 PMC10407731

[ref52] Vitullo M. Grant K. A. Sammarco M. L. Tamburro M. Ripabelli G. Amar C. F. L. (2013). Real-time PCRs assay for serogrouping *Listeria monocytogenes* and differentiation from other *Listeria* spp. Mol. Cell. Probes 27, 68–70. doi: 10.1016/j.mcp.2012.10.001, 23064121

[ref53] Vlachos S. Georgantzis N. (2016). Consumer behaviour towards organic ready-to-eat meals. Int. J. Food Beverage Manuf. Bus. Model. 1, 12–27. doi: 10.4018/IJFBMBM.2016010102

[ref54] Wambogo E. A. Vaudin A. M. Moshfegh A. J. Spungen J. H. van Doren J. M. Sahyoun N. R. (2020). Toward a better understanding of Listeriosis risk among older adults in the United States: characterizing dietary patterns and the sociodemographic and economic attributes of consumers with these patterns. J. Food Prot. 83, 1208–1217. doi: 10.4315/JFP-19-617, 32221521

[ref55] Yin Y. Yao H. Doijad S. Kong S. Shen Y. Cai X. . (2019). A hybrid sub-lineage of *Listeria monocytogenes* comprising hypervirulent isolates. Nat. Commun. 10:4283. doi: 10.1038/s41467-019-12072-1, 31570766 PMC6768887

[ref56] Zendri F. Schmidt V. Mauder N. Loeffler A. Jepson R. E. Isgren C. . (2024). Rapid typing of Klebsiella pneumoniae and *Pseudomonas aeruginosa* by Fourier-transform infrared spectroscopy informs infection control in veterinary settings. Front. Microbiol. 15:1334268. doi: 10.3389/fmicb.2024.1334268, 38371930 PMC10869444

