# Supplementary material for: Fourier transform infrared spectroscopy enables rapid strain typing and cluster analysis of Listeria monocytogenes under diverse growth conditions
Source: Front Microbiol. 2026 Feb 17;17:1735218. doi: 10.3389/fmicb.2026.1735218 (PMC12953571; doi:10.3389/fmicb.2026.1735218)
Supplement: Supplementary file 1 [file Data_Sheet_1.PDF]

Supplementary Tables

Supplementary Table S1. Study strains

Strain ID	Source	Lineage	ST *	CC *	PCR type	WGS Serotype	Serological Serotype	FTIR Serogroup	Reference	Accession number
LL195 [ab]	Vacherin Montd'or cheese	I	ST1	CC1	IVb	4b	4b	4x	Bille, 1990	HF558398
N11-2292	human listeriosis	I	ST1	CC1	IVb	4b	4b	4x	Althaus et al., 2014	JABYYE000000000
N12-0605	Meat / Meat product	I	ST727	CC1	IVb	4b	4b	4x	Ebner et al., 2015	JABYXR000000000
N12-1339	Meat / Meat product	I	ST746	CC1	IVb	4b	4b	4x	Ebner et al., 2015	JABYXM000000000
N12-1996	Milk / Milk product	I	ST1	CC1	IVb	4b	4b	4x	Ebner et al., 2015	JABYXD000000000
N13-0047	Milk / Milk product	I	ST1	CC1	IVb	4b	4b	4x	Ebner et al., 2015	JABYWZ000000000
N11-2747	human listeriosis	I	ST1	CC1	IVb	4b	4b	4x	Althaus et al., 2014	JABYYC000000000
N12-0341	human listeriosis	I	ST1	CC1	IVb	4b	4b	4x	Althaus et al., 2014	JABYXY000000000
N12-0551	human listeriosis	I	ST1	CC1	IVb	4b	4b	4x	Althaus et al., 2014	JABYXU000000000
Scott A	Human listeriosis	I	ST290	CC2	IVb	4b	4b	4x	Fleming et al., 1985	CM001159
N13-1054	human listeriosis	I	ST1285	CC2	IVb	4b	4b	4x	Althaus et al., 2014	QYED00000000
N12-0973 [a]	Meat / Meat product	I	ST2	CC2	IVb	4b	4b	4x	Ebner et al., 2015	JABYXO000000000
N11-1846	Meat / Meat product	I	ST724	CC2	IVb	4b	4b	4x	Ebner et al., 2015	JABYYG000000000
N12-0432	Meat / Meat product	I	ST2	CC2	IVb	4b	4b	4x	Ebner et al., 2015	JABYXX000000000
N12-0466	Meat / Meat product	I	ST2	CC2	IVb	4b	4b	4x	Ebner *et al*., 2015	JABYXW000000000
N14-0435	Milk product	I	ST3	CC3	IIb	1/2b	1/2b	1/2	Ebner et al., 2015	**No WGS**
L94s	Meat / Meat product	I	ST3	CC3	IIb	1/2b	1/2b	1/2	Aalto-Araneda et al., 2020	JABYSW000000000
N2306	Ready-to-eat salads	I	ST4	CC4	IVb	4b	4b	4x	Stephan et al., 2015	CP011004
N14-0487	Plant associated	I	ST4	CC4	IVb	4b	4b	4x	Ebner et al., 2015	**No WGS**
N12-0320	human listeriosis	I	ST4	CC4	IVb	4b	4b	4x	Althaus et al., 2014	JABYXZ000000000

N12-0794	human listeriosis	I	ST4	CC4	IVb	4b	4b	4x	Althaus et al., 2014	JABYXQ000000000
N13-2107	Meat / Meat product	I	ST4	CC4	IVb	4b	4b	4x	Ebner et al., 2015	QYDL00000000
N12-1772	Milk / Milk product	I	ST682	CC4	IVb	4b	4b	4x	Ebner et al., 2015	JABYXH000000000
N11-2675 [ab]	human listeriosis	I	ST1063	CC5	IIb	1/2b	1/2b	1/2	Althaus et al., 2014	QYHF00000000
N16-0044 [ab]	Meat pâté	I	ST6	CC6	IVb	4b	4b	4x	Althaus et al., 2017	CP035187
N12-1387	human listeriosis	I	ST6	CC6	IVb	4b	4b	4x	Althaus et al., 2014	JABYXL000000000
N11-2801	human listeriosis	I	ST6	CC6	IVb	4b	4b	4x	Althaus et al., 2014	JABYYB000000000
N13-1184	Meat / Meat product	I	ST6	CC6	IVb	4b	4b	4x	Ebner et al., 2015	SAMN08995136
N11-1850 [a]	Milk / Milk product	I	ST1290	CC217	IVb	4b	4b	4x	Ebner *et al*., 2015	QYIA00000000
N12-1608 [a]	human listeriosis	I	ST224	CC224	IIb	1/2b	1/2b	1/2	Althaus *et al*., 2014	JABYZR000000000
Lm 45	human listeriosis	I	ST224	CC224	IIb	1/2b	1/2b	1/2	Aalto-Araneda et al., 2020	JABZAC000000000
H34 [ab]	Human listeriosis	I	ST489	CC489	IIb	1/2b	1/2b	1/2	Muchaamba et al., 2018	CP020774
Lm10403S	Human listeriosis	II	ST85	CC7	IIa	1/2a	1/2a	1/2	Edman et al., 1968	NC_017544
N1546 [ab]	Imported cooked ham	II	ST8	CC8	IIa	1/2a	1/2a	1/2	Hächler et al., 2013	CP013724
N11-1617	Meat / Meat product	II	ST8	CC8	IIa	1/2a	1/2a	1/2	Ebner et al., 2015	JABZDM000000000
N11-1649	Meat / Meat product	II	ST743	CC8	IIa	1/2a	1/2a	1/2	Ebner et al., 2015	JABZDL000000000
N11-1584	human listeriosis	II	ST1295	CC8	IIa	1/2a	1/2a	1/2	Althaus et al., 2014	JABZAJ000000000
N11-1346	Human listeriosis	II	ST673	CC8	IIa	1/2a	1/2a	1/2	Althaus *et al*., 2014	JABZDQ000000000
EGDe [a]	Rabbits	II	ST35	CC9	IIc	1/2a	1/2a	1/2	Glaser et al., 2001	NC_003210.1
D:824/5 [ab]	Meat product	II	ST9	CC9	IIc	3c	3c	3	Ebner et al., 2015	**No WGS**
N11-1514 [ab]	Meat / Meat product	II	ST9	CC9	IIc	1/2c	1/2c	1/2	Ebner et al., 2015	JABYZD000000000
N12-1921 [a]	Plant associated	II	ST9	CC9	IIc	1/2c	1/2c	1/2	Ebner et al., 2015	JABYYW000000000
D: 650/8 [ab]	Meat / Meat product	II	ST9	CC9	IIc	3c	3c	3	Ebner et al., 2015	**No WGS**
N12-0152	Milk / Milk product	II	ST9	CC9	IIa	1/2a	1/2a	1/2	Ebner et al., 2015	**No WGS**
N11-1837	human listeriosis	II	ST9	CC9	IIc	1/2c	1/2a	1/2	Althaus et al., 2014	JABZBE000000000

N12-0486	human listeriosis	II	ST9	CC9	IIc	1/2c	1/2c	1/2	Althaus et al., 2014	JABYZC000000000
N13-0001 [a]	human listeriosis	II	ST9	CC9	IIc	1/2c	1/2c	1/2	Althaus et al., 2014	JABYYS000000000
HE161E/1	Processing environment	II	ST9	CC9	IIc	1/2c	1/2c	1/2	Wambui et al., 2020	JABYSI010000000
Lm 22/3A	Human listeriosis	II	ST9	CC9	IIc	1/2c	1/2c	1/2	Wambui et al., 2020	JABYZG010000000
L51s	Meat / Meat product	II	ST9	CC9	IIc	1/2c	1/2c	1/2	Wambui et al., 2020	JABYSB000000000
N12-1864	Milk / Milk product	II	ST9	CC9	IIa	1/2a	1/2a	1/2	Ebner et al., 2015	**No WGS**
Lm3136 [ab]	Tomme cheese	II	ST18	CC18	IIa	1/2a	1/2a	1/2	Bille et al., 2006	CP013723
N11-2183	Plant associated	II	ST20	CC20	IIa	1/2a	1/2a	1/2	Ebner et al., 2015	JABZDF000000000
Lm3163	Tomme cheese	II	ST26	CC26	IIa	1/2a	1/2a	1/2	Bille et al., 2006	CP013722
N11-1515	Milk product	II	ST29	CC29	IIa	1/2a	1/2a	1/2	Ebner et al., 2015	JABZDO000000000
N05-195 [c]	Meat / Meat product	II	ST31	CC31	IIa	1/2a	1/2a	1/2	Ebner et al., 2015	QYIT00000000
N11-1905	Meat / Meat product	II	ST121	CC121	IIa	1/2a	1/2a	1/2	Ebner *et al.*, 2015	JABZDG000000000
N12-1024	Meat / Meat product	II	ST121	CC121	IIa	1/2a	1/2a	1/2	Ebner *et al.*, 2015	JABZCE000000000
N13-0119	human listeriosis	II	ST121	CC121	IIa	1/2a	1/2a	1/2	Althaus *et al.*, 2014	JABZAK000000000
N12-0367	human listeriosis	II	ST121	CC121	IIa	1/2a	1/2a	1/2	Althaus *et al.*, 2014	JABZAE000000000
N842_15	Human prosthetic joint	II	ST412	CC412	IIa	1/2a	3a	3	Muchaamba et al., 2020	CP046361
N843_10	Human prosthetic joint	II	ST412	CC412	IIa	1/2a	3a	3	Muchaamba et al., 2020	CP046362
N12-1273	Human listeriosis	II	ST412	CC412	IIa	1/2a	1/2a	1/2	Althaus *et al.*, 2014	QYFZ00000000
WSLC1019 [a]	Animal isolate	III	ST130	CC69	L	4c	4c	4x	ATCC 19116	CP013286
LMNC318 [ab]	Ruminant listeriosis	III	ST70	CC70	L	4a/4c	4a/4c	4x	Dreyer et al., 2016	ERS1324346
LMNC326 [ab]	Ruminant listeriosis	III	ST70	CC70	L	4a/4c	4a/4c	4x	Dreyer et al., 2016	ERS1324347
WLSC1020 [a]	Animal isolate	III	ST71	CC131	L	4a	4a	4x	ATCC 19114	NZ_CP013287
F2365	Genome control	I	ST1	CC1	IVb	4b	4b	4x	Nelson et al., 2004	NC_002973
HE1E	Environment	II	ST101	CC101	IIa	1/2a	1/2a	1/2	Wambui et al., 2020	SAMN15368389
LE55E	Environment	II	ST101	CC101	IIa	1/2a	1/2a	1/2	Wambui et al., 2020	JBGMUS000000000

RE46E	Environment	II	ST101	CC101	IIa	1/2a	1/2a	1/2	Wambui et al., 2020	SAMN15368393
LL66/3	Wild bird, feces	II	ST101	CC101	IIa	1/2a	1/2a	1/2	Wambui et al., 2020	JABYTE000000000
N12-0258	Human	II	ST681	CC101	IIa	1/2a	1/2a	1/2	Althaus *et al*., 2014	QYGZ00000000
TT107E	Environment	II	ST101	CC101	IIa	1/2a	1/2a	1/2	Wambui et al., 2020	SAMN15368393
LU74/1	Meat	II	ST101	CC101	IIa	1/2a	1/2a	1/2	Wambui et al., 2020	JABYVL000000000
HT45E	Poultry	II	ST155	CC155	IIa	1/2a	1/2a	1/2	Wambui et al., 2020	JABYWL000000000
TT82E	Fish	II	ST155	CC155	IIa	1/2a	1/2a	1/2	Wambui et al., 2020	JABYWO000000000
LK132 [ab]	Fish	II	ST155	CC155	IIa	1/2a	1/2a	1/2	Wambui et al., 2020	JABYWK000000000
N12-0869 [a]	Human	I	ST4	CC4	IVb	4b	4b	4x	Wambui et al., 2020	JABYXP000000000
JHM270	Cow, feces	I	ST2	CC2	IVb	4b	4b	4x	Wambui et al., 2020	JABYRO000000000
N12-1859	Food	I	ST2	CC2	IVb	4b	4b	4x	Wambui et al., 2020	JABYXF000000000
N11-1251 [a]	Sausage	I	ST59	CC59	IIb	1/2b	1/2b	1/2	Wambui et al., 2020	JABYZV000000000
N12-2449	Milk	I	ST59	CC59	IIb	1/2b	1/2b	1/2	Wambui et al., 2020	JABYZL000000000
LmE188 [a]	Meat	I	ST59	CC59	IIb	1/2b	1/2b	1/2	Wambui et al., 2020	JABYZZ000000000
LL31/1	Vegetables and salads	I	ST59	CC59	IIb	1/2b	1/2b	1/2	Aalto-Araneda et al., 2020	JABYSR000000000
N12-0563 [ab]	Meat	II	ST9	CC9	IIc	1/2c	1/2c	1/2	Wambui et al., 2020	JABYZB000000000
N12-2151 [a]	Poultry	II	ST9	CC9	IIc	1/2c	1/2c	1/2	Wambui et al., 2020	JABYYV000000000
N12-2271	Meat	II	ST9	CC9	IIc	1/2c	1/2c	1/2	Wambui et al., 2020	JABYYU000000000
N12-2386	Environment	II	ST9	CC9	IIc	1/2c	1/2c	1/2	Wambui et al., 2020	JABYYT000000000
Lm 72	Human	I	ST6	CC6	IVb	4b	4b	4x	Wambui et al., 2020	JABYYO000000000
LmE162	Meat	I	ST6	CC6	IVb	4b	4b	4x	Wambui et al., 2020	JABYYH000000000
LmS1 [a]	Environment	II	ST121	CC121	IIa	1/2a	1/2a	1/2	Wambui et al., 2020	JABZDV000000000
N11-1218	Poultry	II	ST121	CC121	IIa	1/2a	1/2a	1/2	Wambui et al., 2020	JABZDT000000000
LM86	Fish	II	ST155	CC155	IIa	1/2a	1/2a	1/2	Wambui et al., 2020	JABYWR000000000
Lm 69	Human	II	ST155	CC155	IIa	1/2a	1/2a	1/2	Wambui et al., 2020	JABZDY000000000
N13-0771	Human	II	ST155	CC155	IIa	1/2a	1/2a	1/2	Aalto-Araneda et al., 2020	NZ_QYEL00000000

N14-0322	Meat	II	ST121	CC121	IIc	3c	3c	3	Ebner et al., 2015	NZ_QYDH00000000
N12-0318 [a]	Qourn	II	ST204	CC204	IIa	3a	3a	3	Wambui et al., 2020	JABYYR000000000
E7 [ab]	Dairy	II	ST101	CC101	IIa	3a	3a	3	Wambui et al., 2020	SAMN15368509
LK42/1 [a]	Fish	II	ST7	CC7	IIa	3a	3a	3	Wambui et al., 2020	SAMN15368510
LK55/1 [ab]	Fish	II	ST18	CC18	IIa	3a	3a	3	Wambui et al., 2020	SAMN15368511
LM128 [a]	Vegetable	II	nd	nd	IIa	3a	3a	3	Aalto-Araneda et al., 2020	**No WGS**
N14-0261 [a]	Meat	II	ST9	CC9	IIc	1/2c	1/2c	1/2	Horlbog et al. 2018	NZ_QELW00000000

[a] Subset of strains subcultured on eight different media and incubated at 37°C for 24 h. [b] Subset of strains subcultured on either Blood agar or ALOA agar and incubated at 37°C for 20 or 48 h. [c] Also known as N14-195. * **ST**: sequence type; **CC**: clonal complex. **No WGS**: not sequenced. **nd**: not determined.

Supplementary Table S2. Strains used in the blinded outbreak investigation study

Blinded study code	Strain ID	Source	Lineage	ST *	cgMLST	CC *	PCR type	WGS Serotype	FTIR Serogroup	Reference	Accession number
F1	N22-0963	Control	II	ST9	22911	CC9	IIc	1/2c	1/2	This study	JBNVVJ000000000
F2	N21-1040	Control	II	ST9	7780	CC9	IIc	1/2c	1/2	This study	JBNVVI000000000
F3	N22-3358	Outbreak 1	II	ST9	22912	CC9	IIc	1/2c	1/2	This study	JBNVVH000000000
F7	N22-3360	Outbreak 1	II	ST9	22912	CC9	IIc	1/2c	1/2	This study	JBNVVG000000000
F9	N23-0051	Outbreak 1	II	ST9	22912	CC9	IIc	1/2c	1/2	This study	JBNVVF000000000
F4	N21-0849	Outbreak 2	II	ST9	4838	CC9	IIc	1/2c	1/2	This study	JBNVVE000000000
F8	N21-1041	Outbreak 2	II	ST9	4838	CC9	IIc	1/2c	1/2	This study	JBNVVD000000000
F11	N21-1044	Outbreak 2	II	ST9	4838	CC9	IIc	1/2c	1/2	This study	JBNVVC000000000
F5	N21-1451	Outbreak 3	II	ST9	22644	CC9	IIc	1/2c	1/2	This study	JBNVVB000000000
F6	N22-1391	Outbreak 3	II	ST9	22644	CC9	IIc	1/2c	1/2	This study	JBNVVA000000000
F10	N22-1823	Outbreak 3	II	ST9	22644	CC9	IIc	1/2c	1/2	This study	JBNVUZ000000000
F15	N22-1825	Outbreak 3	II	ST9	22644	CC9	IIc	1/2c	1/2	This study	JBNVUY000000000

F12	N22-3271	Control	II	ST9	18775	CC9	IIc	1/2c	1/2	This study	JBNVUX000000000
F13	N22-3621	Control	II	ST9	4689	CC9	IIc	1/2c	1/2	This study	JBNVUW000000000
F14	N22-3638	Control	II	ST9	7887	CC9	IIc	1/2c	1/2	This study	JBNVUV000000000

* **ST**: sequence type; **CC**: clonal complex.

Supplementary Table S3. Comparison of FTIR-based classification on Blood agar and BHI agar

	Blood agar	BHI agar
**Accuracy**	100% (854/854)	100% (860/860)
**Green***	93.6% (799/854)	92.8% (798/860)
**Yellow***	6.4% (55/854)	7.2% (62/860)
**Red***	0/854	0/860

*Confidence scores of FTIR-based classification for 103 *L. monocytogenes* strains grown on either Blood agar or BHI agar at 37ºC for 24 h. Shown are the percentages of spectra for each media type, classified as green, orange, or red, relative to the total number of spectra per media, from a minimum of two biological replicates.

Supplementary Table S4. Comparison of FTIR-based classification across media

	Blood agar	BHI agar	BHI broth	TSA	ALOA	Palcam	Rapid'Lmono	Oxford
**Accuracy**	100% (394/394)	100% (283/283)	100% (369/369)	100% (251/251)	100% (240/240)	100% (265/265)	100% (307/307)	100% (230/230)
**Green***	97.0% (382/394)	92.9% (263/283)	88.3% (326/369)	87.3% (219/251)	99.2% (238/240)	92.8% (246/265)	68.7% (211/307)	95.7% (220/230)
**Yellow***	3.0% (12/394)	7.1% (20/283)	11.7% (43/369)	12.7% (32/251)	0.8% (2/240)	7.2% (19/265)	31.3% (96/307)	4.3% (10/230)
**Red***	0/394	0/283	0/369	0/251	0/240	0/265	0/307	0/230

*Confidence scores of FTIR-based classification for 32 *L. monocytogenes* isolates grown on different media at 37ºC for 24 h. Shown are the percentages of spectra for each media type, classified as green, orange, or red, relative to the total number of spectra per media, from a minimum of two biological replicates.

Supplementary Table S5. Comparison of FTIR-based classification across media and temperature

Media	Blood agar		ALOA		Rapid'Lmono		Oxford	
Temperature (ºC)	25ºC	37ºC	25ºC	37ºC	25ºC	37ºC	25ºC	37ºC
Accuracy	100% (200/200)	100% (394/394)	100% (266/266)	100% (240/240)	100% (278/278)	100% (307/307)	100% (274/274)	100% (230/230)
Green*	66.5% (133/200)	97.0% (382/394)	88.0% (234/266)	99.2% (238/240)	46% (128/278)	68.7% (211/307)	78.8% (216/274)	95.7% (220/230)
Yellow*	33.5% (67/200)	3.0% (12/394)	12.0% (32/266)	0.8% (2/240)	54% (150/278)	31.3% (96/307)	21.2% (58/274)	4.3% (10/230)
Red*	0/200	0/394	0/266	0/240	0/278	0/307	0/274	0/230

*Confidence scores of FTIR-based classification for 32 *L. monocytogenes* isolates grown for 24 h on different media and temperature. Shown are the percentages of spectra for each media type, classified as green, orange, or red, relative to the total number of spectra per media, from a minimum of two biological replicates.

Supplementary Table S6. Comparison of FTIR-based classification across media and incubation duration

Media	Blood agar		ALOA	
Incubation time	24 h	48 h	24 h	48 h
Accuracy	100% (87/87)	100% (100/100)	100% (94/94)	100% (138/138)
Green*	96.6% (84/87)	90% (90/100)	100% (94/94)	94.2% (130/138)
Yellow*	3.4% (3/87)	10% (10/100)	0/94	5.8% (8/138)
Red*	0/87	0/100	0/94	0/138

*Confidence scores of FTIR-based classification for 15 *L. monocytogenes* isolates grown at 37°C on different media and incubation durations. Shown are the percentages of spectra for each media type, classified as green, orange, or red, relative to the total number of spectra per media, from a minimum of two biological replicates.

References

1. Aalto-Araneda M, Pöntinen A, Pesonen M, Corander J, Markkula A, Tasara T, Stephan R, Korkeala H. Strain Variability of *Listeria monocytogenes* under NaCl Stress Elucidated by a High-Throughput Microbial Growth Data Assembly and Analysis Protocol. Appl Environ Microbiol. 2020 Mar 2;86(6):e02378-19. doi: 10.1128/AEM.02378-19.
2. Althaus D, Lehner A, Brisse S, Maury M, Tasara T, and Stephan R. 2014. Characterization of *Listeria monocytogenes* strains isolated during 2011-2013 from human infections in Switzerland. Foodborne. Pathog. Dis; 11: 753-758. doi: 10.1089/fpd.2014.1747.
3. Althaus, D.; Jermini, M.; Giannini, P.; Martinetti, G.; Reinholz, D.; Nüesch-Inderbinen, M.; Lehner, A.; Stephan, R. Local Outbreak of *Listeria monocytogenes* Serotype 4b Sequence Type 6 due to Contaminated Meat Pâté. Foodborne Pathog. Dis. 2017, 14, 219–222.
4. Bille J. 1990. Epidemiology of human listeriosis in Europe with special reference to the Swiss outbreak, p 71-74 In Miller AJ, Smith JL, Somkuti GA, editors. Foodborne listeriosis. Elsevier, New York.
5. Bille, J.; Blanc, D.S.; Schmid, H.; Boubaker, K.; Baumgartner, A.; Siegrist, H.H.; Tritten, M.L.; Lienhard, R.; Berner, D.; Anderau, R.; et al. Outbreak of human listeriosis associated with tomme cheese in northwest Switzerland, 2005. Eurosurveillance 2006, 11, 11–12.
6. Dreyer, M.; Aguilar-Bultet, L.; Rupp, S.; Guldimann, C.; Stephan, R.; Schock, A.; Otter, A.; Schüpbach, G.; Brisse, S.; Lecuit, M.; et al. *Listeria monocytogenes* sequence type 1 is predominant in ruminant rhombencephalitis. Sci. Rep. 2016, 6, 36419.
7. Ebner R, Stephan R, Althaus D, Brisse S, Maury M, and Tasara T. 2015. Phenotypic and genotypic characteristics of *Listeria monocytogenes* strains isolated during 2011-2014 from different food matrices in Switzerland. Food Control; 57: 321-326. doi: 10.1016/j.foodcont.2015.04.030
8. Edman, D.C.; Pollock, M.B.; Hall, E.R. *Listeria monocytogenes* L Forms I. Induction, Maintenance, and Biological Characteristics1. J. Bacteriol. 1968, 96, 352–357.
9. Fleming, D.W.; Holmes, M.B.; Audurier, A.; Cochi, S.L.; Macdonald, K.L.; Brondum, J.; Hayes, P.S.; Plikaytis, B.D.; Broome, C.V.; Reingold, A.L. Pasteurized Milk as a Vehicle of Infection in an Outbreak of Listeriosis. N. Engl. J. Med. 1985, 312, 404–407.
10. Glaser, P.; Frangeul, L.; Buchrieser, C.; Rusniok, C.; Amend, A.; Baquero, F.; Berche, P.; Bloecker, H.; Brandt, P.; Chakraborty, T.; et al. Comparative genomics of *Listeria* species. Science 2001, 294, 849–852.
11. Guldimann C, Bärtschi1 M, Frey J, Zurbriggen A, Seuberlich T, and Oevermann A. 2015. Increased spread and replication efficiency of *Listeria monocytogenes* in organotypic brain-slices is related to multilocus variable number of tandem repeat analysis (MLVA) complex. BMC Microbiol; 15: 134. doi: 10.1186/s12866-015-0454-0.
12. Hächler, H.; Marti, G.; Giannini, P.; Lehner, A.; Jost, M.; Beck, J.; Weiss, F.; Bally, B.; Jermini, M.; Stephan, R.; et al. Outbreak of Listerosis due to Imported Cooked Ham, Switzerland 2011. Eurosurveillance 2013, 18. Available online: http://www.eurosurveillance.org/ViewArticle.aspx?ArticleId=20469 (accessed on 11 August 2020).
13. Horlbog JA, Jang H, Gopinath G, Stephan R, Guldimann C. Whole-Genome Sequences of Six *Listeria monocytogenes* Strains Isolated from Food. Microbiol Resour Announc. 2018 Oct 11;7(14):e01036-18. doi: 10.1128/MRA.01036-18.
14. Muchaamba, F.; Eshwar, A.K.; Von Ah, U.; Stevens, M.J.A.; Tasara, T. Evolution of *Listeria monocytogenes* During a Persistent Human Prosthetic Hip Joint Infection. Front. Microbiol. 2020, 11, 1726

15. Muchaamba, F.; Guldimann, C.; Tasara, T.; Mota, M.I.; Braga, V.; Varela, G.; Algorta, G.; Klumpp, J.; Jermini, M.; Stephan, R. Full-Genome Sequence of *Listeria monocytogenes* Strain H34, Isolated from a Newborn with Sepsis in Uruguay. Genome Announc. 2017, 5, e00544-17.
16. Nelson KE, Fouts DE, Mongodin EF, Ravel J, DeBoy RT, Kolonay JF, Rasko DA, Angiuoli SV, Gill SR, et al. 2004. Whole genome comparisons of serotype 4b and 1/2a strains of the food-borne pathogen *Listeria monocytogenes* reveal new insights into the core genome components of this species. Nucleic Acids Res. Apr 28;32(8):2386-95. doi: 10.1093/nar/gkh562.
17. Stephan R, Althaus D, Kiefer S, Lehner A, Hatz C, Schmutz C, Jost M, Gerber N, Baumgartner A, Hächler, H, et al. Foodborne transmission of *Listeria monocytogenes* via ready-to-eat salad: A nationwide outbreak in Switzerland, 2013–2014. Food Control 2015, 57, 14–17.
18. Tasara T, Ebner R, Klumpp J, Stephan R. 2015. Complete genome sequence of *Listeria monocytogenes* N2306, a strain associated with the 2013-2014 listeriosis outbreak in Switzerland. Genome Announc 3(3):e00553-15. doi:10.1128/genomeA.00553-15.
19. Tasara T, Klumpp J, Bille J, Stephan R. 2016. Genome sequences of *Listeria monocytogenes* strains responsible for cheese- and cooked ham product-associated Swiss listeriosis outbreaks in 2005 and 2011. Genome Announc 4(2):e00106-16. doi:10.1128/genomeA.00106-16.
20. Wambui J, Eshwar AK, Aalto-Araneda M, Pöntinen A, Stevens MJA, Njage PMK, Tasara T. The Analysis of Field Strains Isolated From Food, Animal and Clinical Sources Uncovers Natural Mutations in *Listeria monocytogenes* Nisin Resistance Genes. Front Microbiol. 2020 Oct 6;11:549531. doi: 10.3389/fmicb.2020.549531.
