# Supplementary material for: Fourier transform infrared spectroscopy enables rapid strain typing and cluster analysis of Listeria monocytogenes under diverse growth conditions
Source: Front Microbiol. 2026 Feb 17;17:1735218. doi: 10.3389/fmicb.2026.1735218 (PMC12953571; doi:10.3389/fmicb.2026.1735218)
Supplement: Supplementary file 2 [file Data_Sheet_2.PDF]

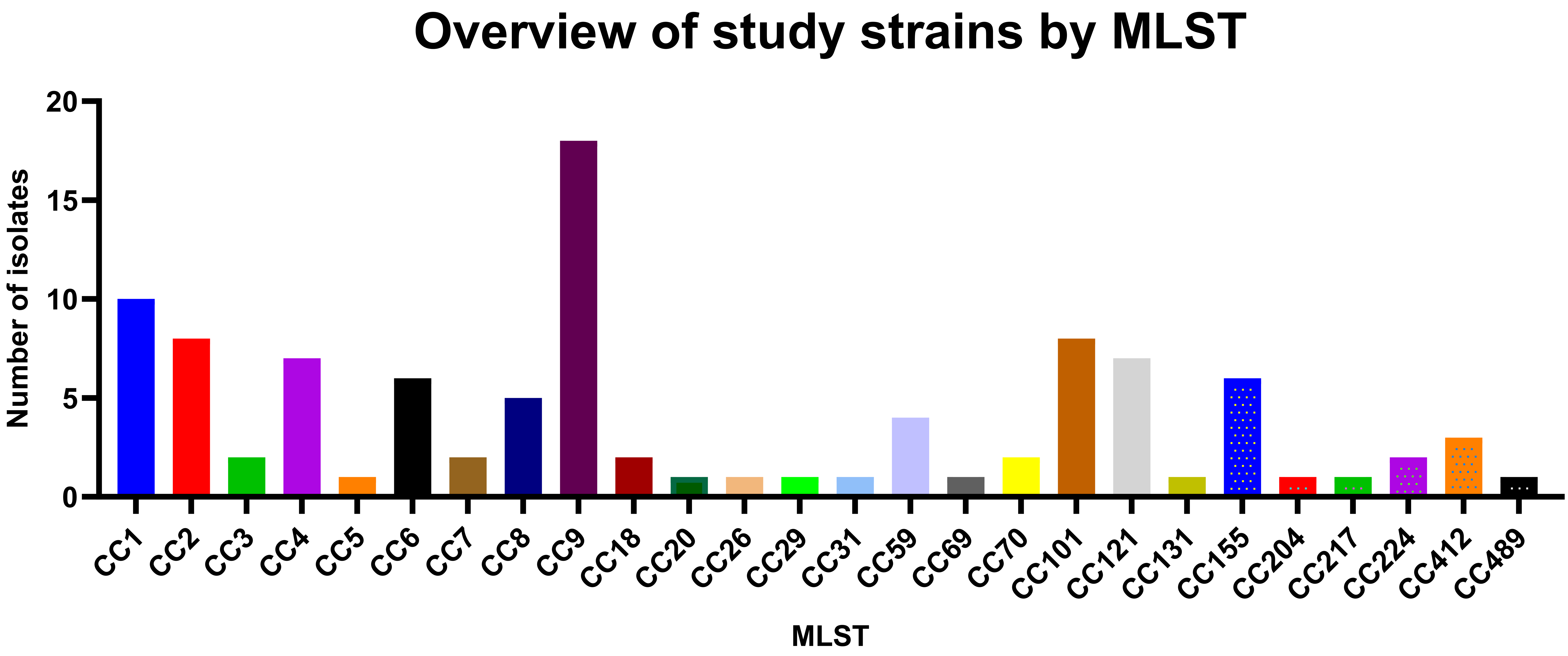

Supplementary Figure S1. An overview of distribution of *L. monocytogenes* strains used in this study based on their MLST clonal complexes. The graph excludes CC9 isolates (n=15) used in the blinded outbreak investigation study.

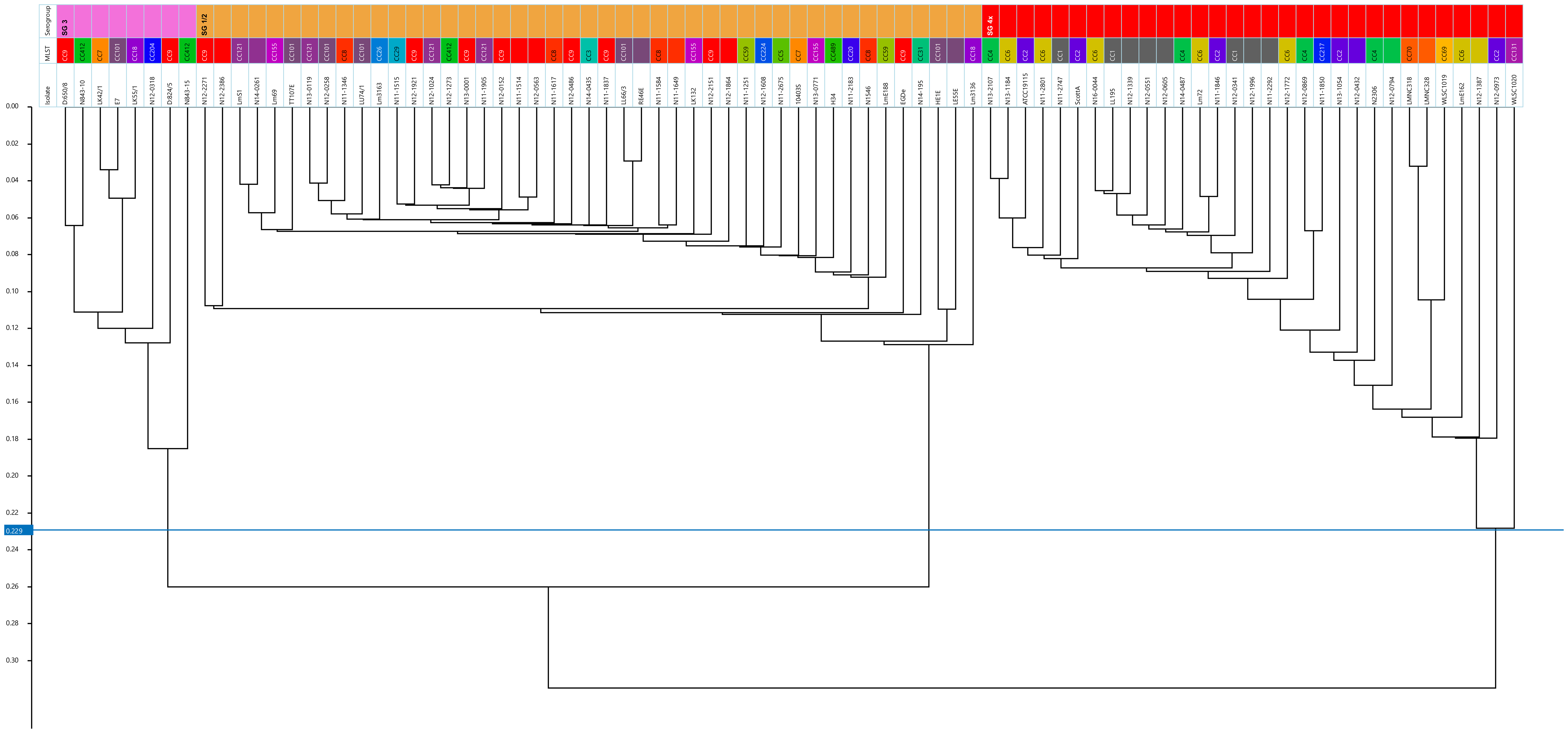

Supplementary Figure S2. Dendrogram illustrating clustering of FTIR spectra of *L. monocytogenes* strains according to MLST CC. Clustering analysis was based on the average spectra per isolate, derived from at least two independent biological replicates, of cultures grown on Blood agar at 37°C for 24 h, each measured in triplicate. The vertical blue line marks the cut-off value for clustering (0.229). Dimensionality reduction was performed using PCA (12 PCs / 95.5% variance). The analysis was based on the 1300–800 cm^{-1} spectral window, corresponding to polysaccharides.

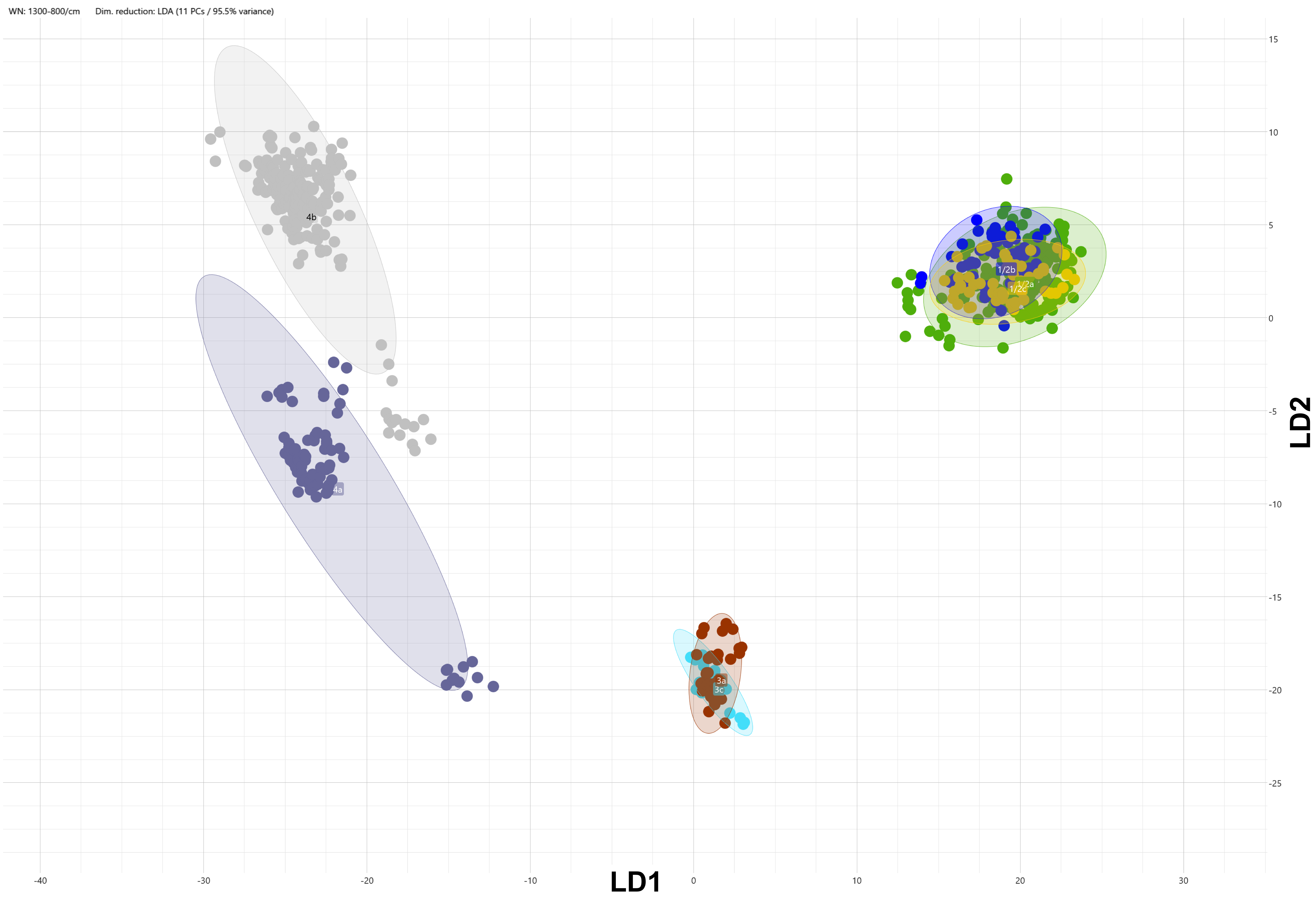

Supplementary Figure S3. Scatter plots from clustering FTIR spectra of *L. monocytogenes* strains according to serotype. Spectra are color-coded by serotype (green: 1/2a: blue: 1/2b, yellow: 1/2c, brown: 3a, cyan 3c, purple: 4a/c, grey: 4b). Each strain is represented by at least six spectra, with each symbol (•) corresponding to a technical replicate from at least two independent biological repeats. Dimensionality reduction was performed using LDA. Wave number region 1300–800 cm^{-1} (polysaccharides) of spectra derived from cultures grown on Blood agar at 37°C for 24 h was analysed .

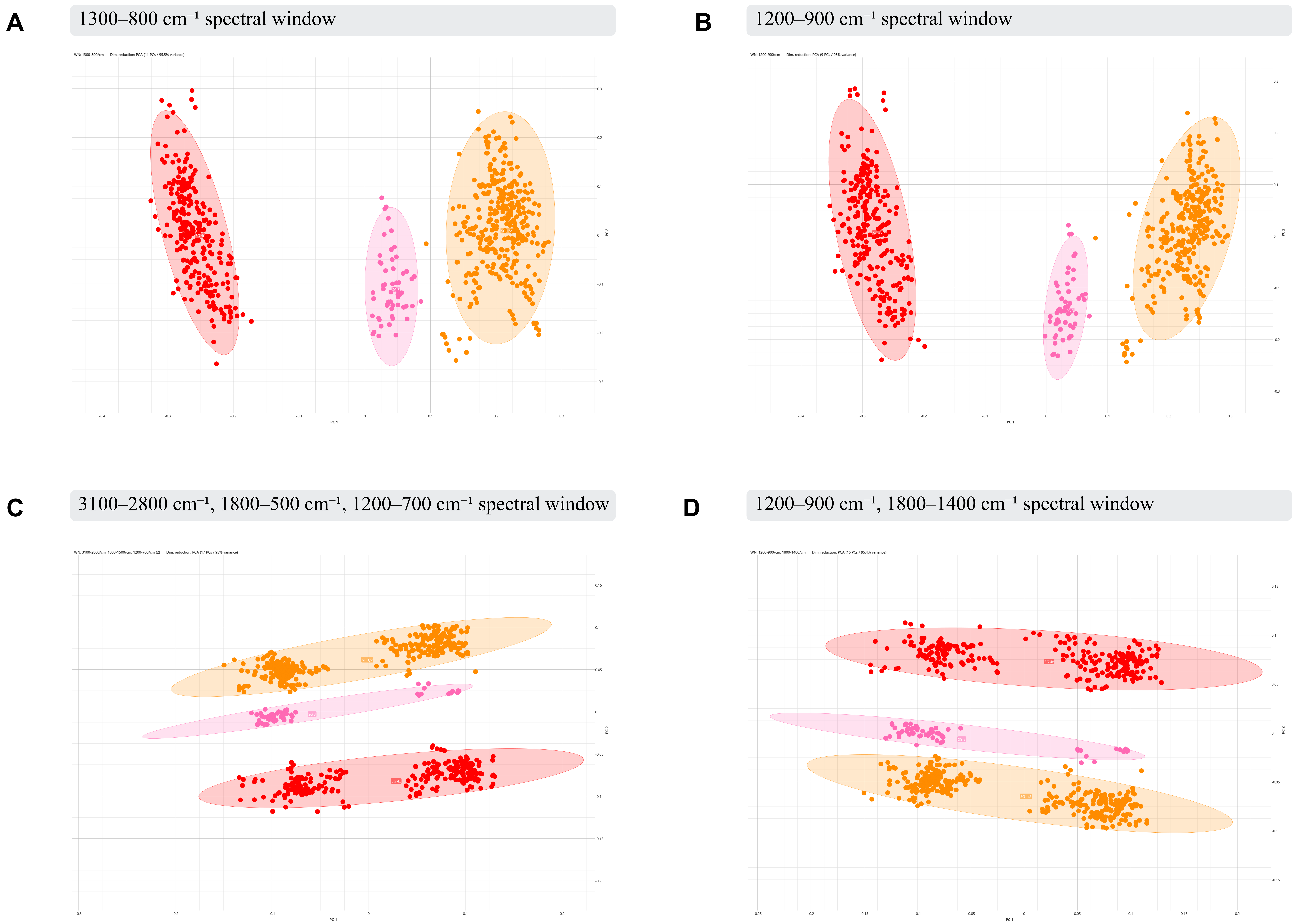

Supplementary Figure S4. Scatter plots from clustering FTIR spectra of *L. monocytogenes* strains according to serogroup. Spectra are color-coded by serogroup (orange: 1/2: pink: 3, red: 4). Each strain is represented by at least six spectra, with data analysed using PCA. The scatter plots display the first two principal components (PC) axes. (**A**) Default spectra window:1300–800 cm^{-1}, (**B**) Polysaccharides spectral window 1200–900 cm^{-1} (**C**) Spectral window adapted from Rebuffo et al., 2006 (**D**) Spectral window adapted from Rebuffo-Scheer et al., 2007.

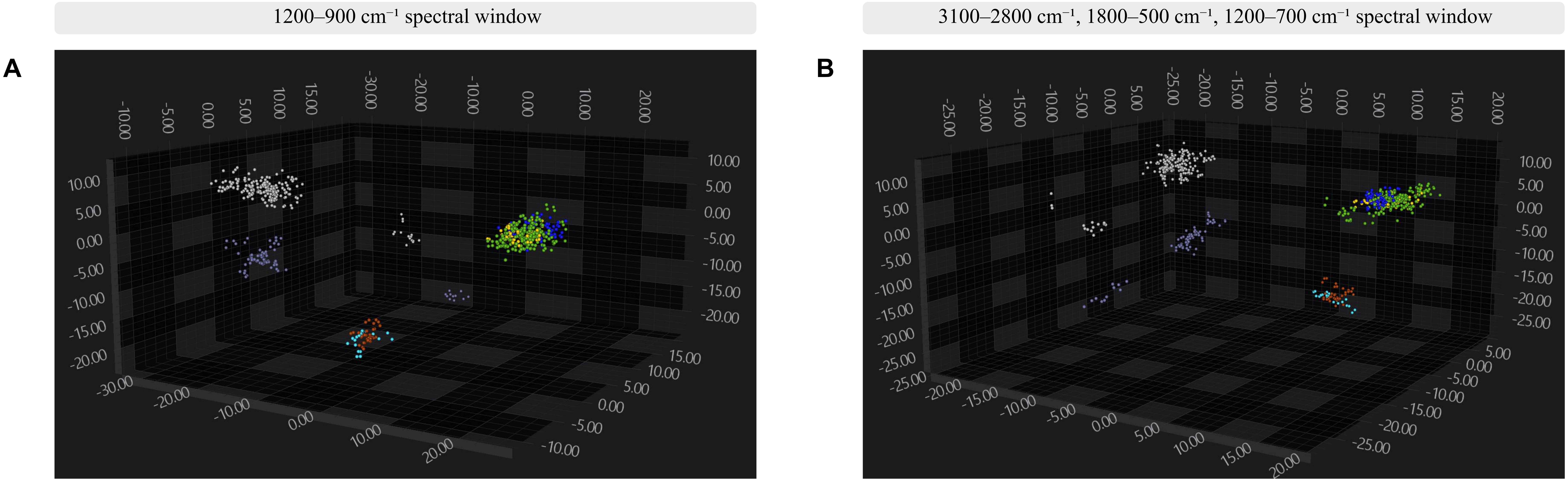

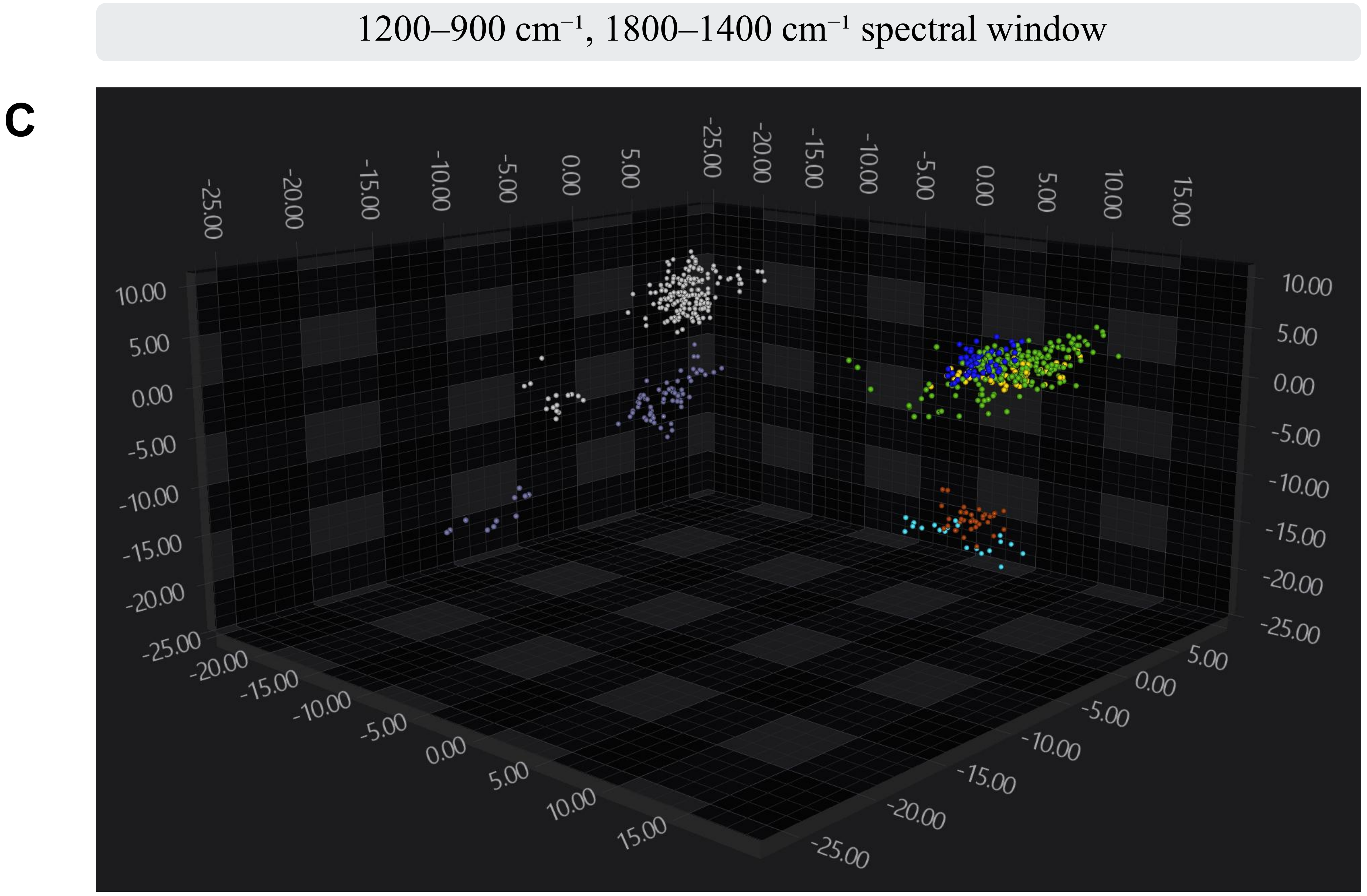

Supplementary Figure S5. Clustering of *L. monocytogenes* strains based on serotype. 3D scatter plot showing the distribution of *L. monocytogenes* strains within the FTIR spectral space. The plot displays the first three LD axes, with spectra color-coded by serotype (green: 1/2a: blue: 1/2b, yellow: 1/2c, brown: 3a, cyan 3c, purple: 4a/c, grey: 4b). Each isolate is represented by at least six spectra, with each symbol (•) corresponding to a technical replicate from at least two independent biological repeats. Dimensionality reduction was performed using LDA. (**A**) Polysaccharides spectral window 1200–900 cm^{-1} (**B**) Spectral window adapted from Rebuffo et al., 2006 (**C**) Spectral window adapted from Rebuffo-Scheer et al., 2007.

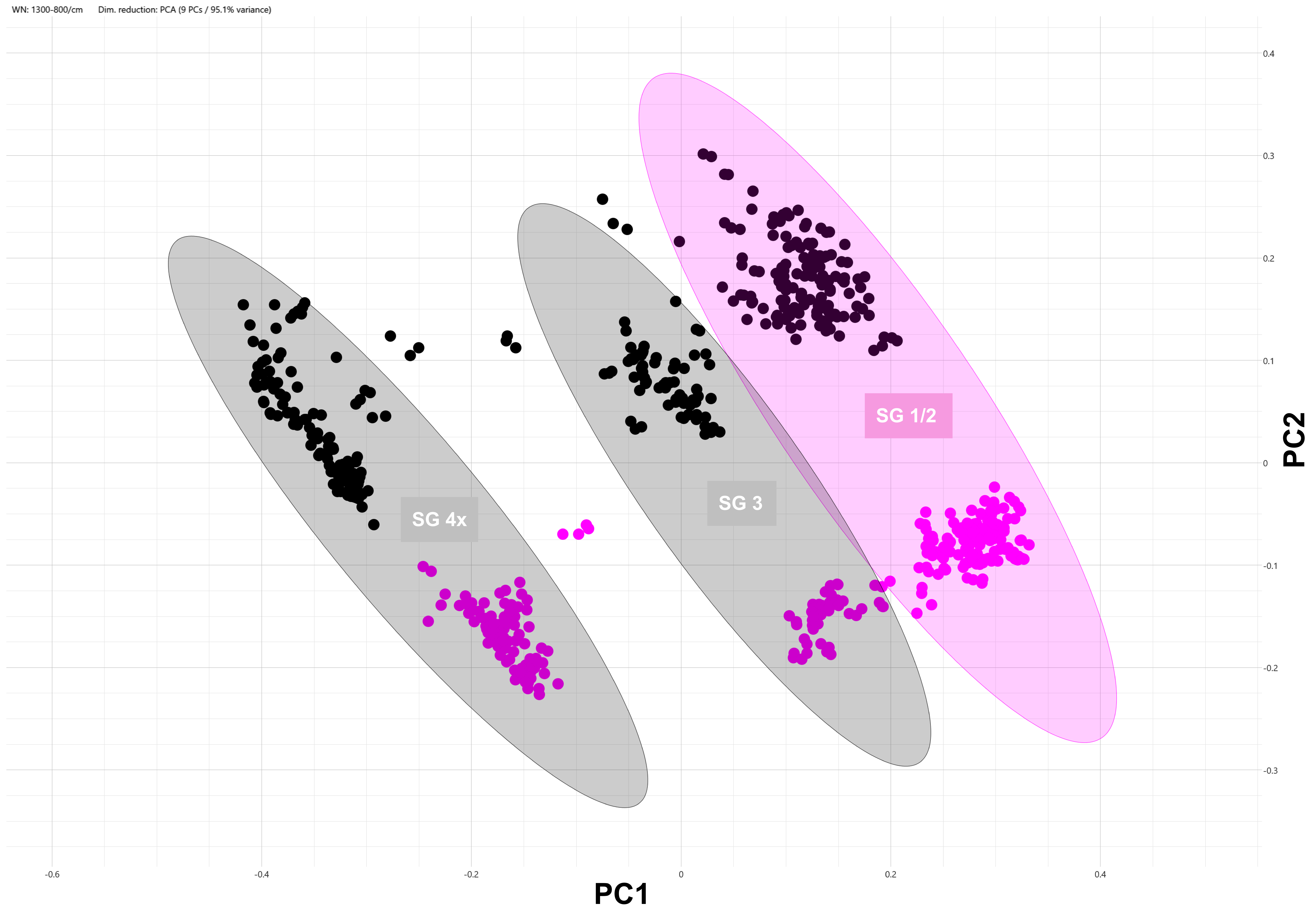

Supplementary Figure S6. Growth media strongly influences the FTIR spectral profiles of *L. monocytogenes* strains. Data are shown as a 2D scatter plot of the FTIR spectra from 32 representative *L. monocytogenes* strains grown on Blood agar and Rapid'Lmono at 37°C for 24 h. Each isolate is represented by at least six spectra, with each symbol (•) corresponding to a technical replicate from at least two independent biological repeats. The symbols are color-coded by media, Blood agar (**magenta**) and Rapid'Lmono (**black**). The 95% confidence ellipses of sample spectral data denote the serogroups (SG). The analysis was based on the 1300–800 cm^{-1} spectral window. Dimensionality reduction was performed using PCA with 9 PCs, capturing 95.1% of the variance. The plot displays the first two PC axes.

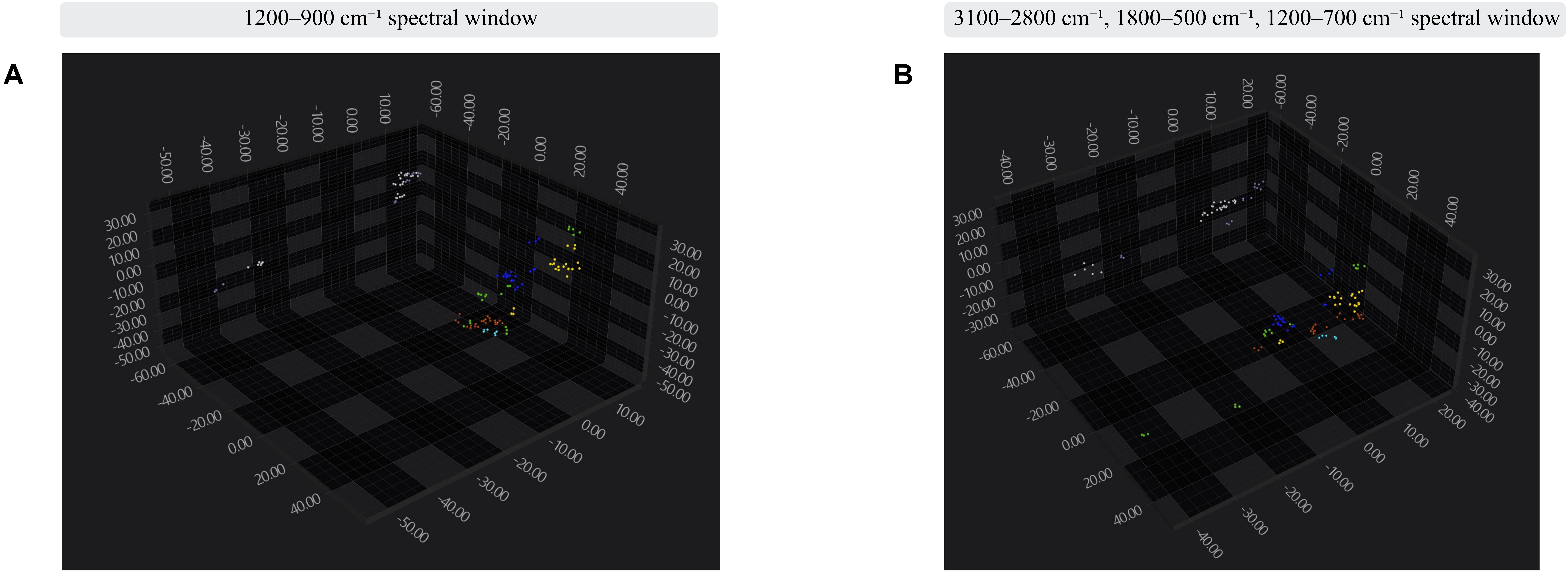

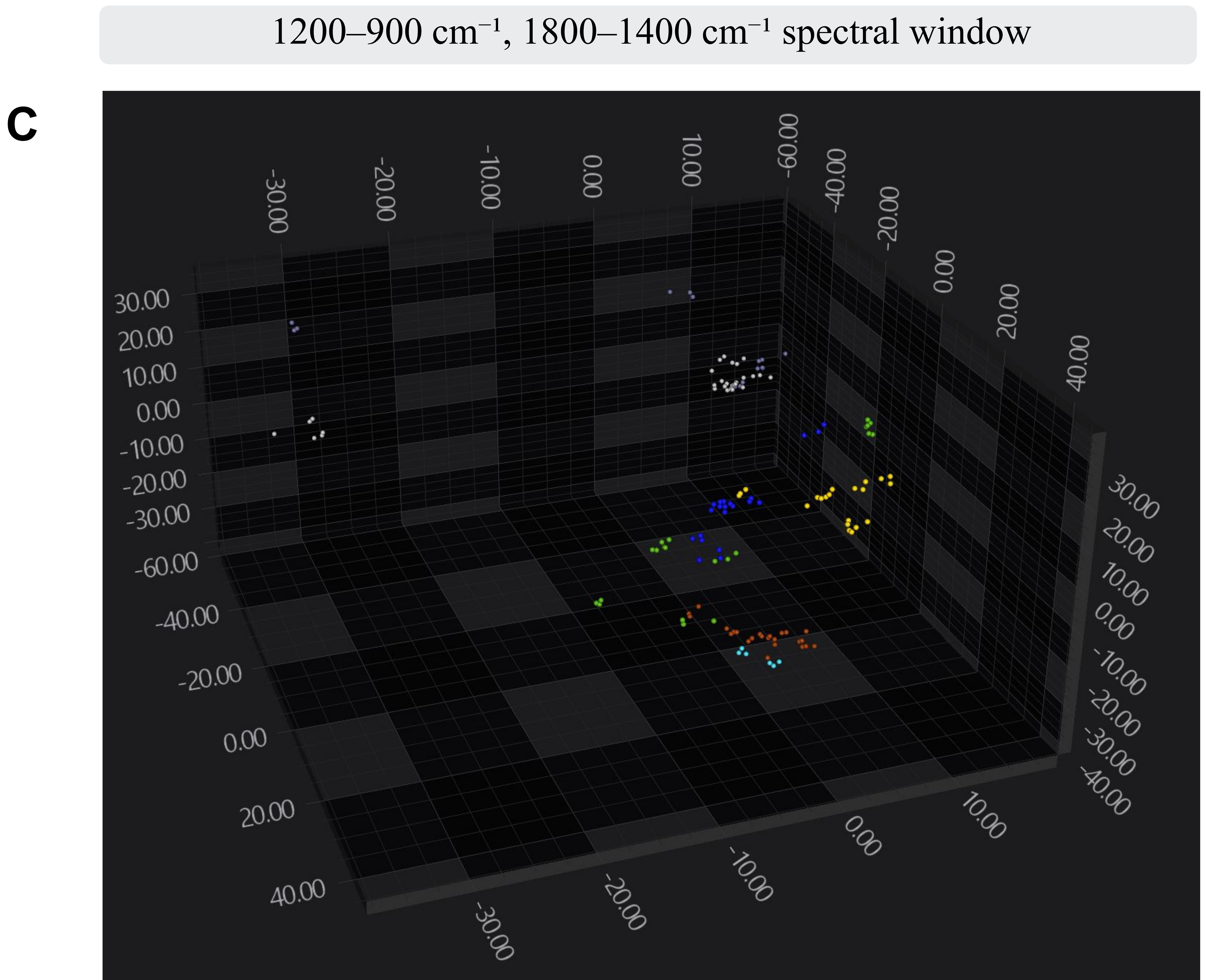

Supplementary Figure S7. Clustering of *L. monocytogenes* strains based on serotype. 3D scatter plot showing the distribution of *L. monocytogenes* strains within the FTIR spectral space. The plot displays the first three LD axes, with spectra color-coded by serotype (green: 1/2a: blue: 1/2b, yellow: 1/2c, brown: 3a, cyan 3c, purple: 4a/c, grey: 4b). Each isolate is represented by at least six spectra, with each symbol (•) corresponding to a technical replicate from at least two independent biological repeats. All cultures were grown on Rapid'Lmono agar at 25°C for 24 h. Dimensionality reduction was performed using LDA. (**A**) Polysaccharides spectral window 1200–900 cm^{-1} (**B**) Spectral window adapted from Rebuffo et al., 2006 (**C**) Spectral window adapted from Rebuffo-Scheer et al., 2007.

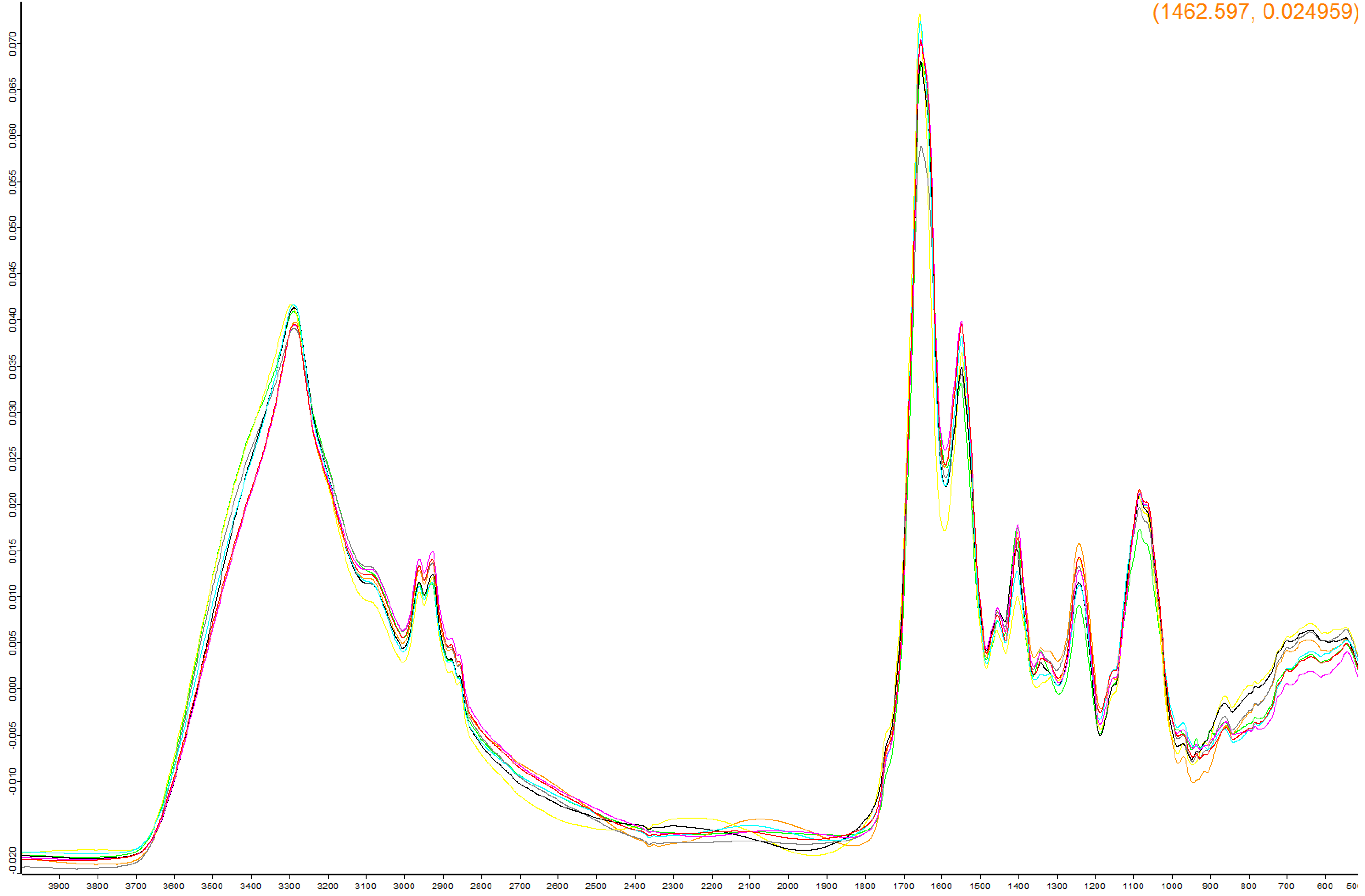

Supplementary Figure S8. Representative vector-normalized spectra of *L. monocytogenes* strain LL195, generated using OPUS software, are shown for visualization purposes. The strain was cultivated at 37 °C for 24 h on various media: ALOA (green), Blood agar (red), Oxford agar (grey), Palcam agar (purple), BHI agar (brown), BHI broth (orange), TSA (yellow), and RAPID'L.mono agar (black).

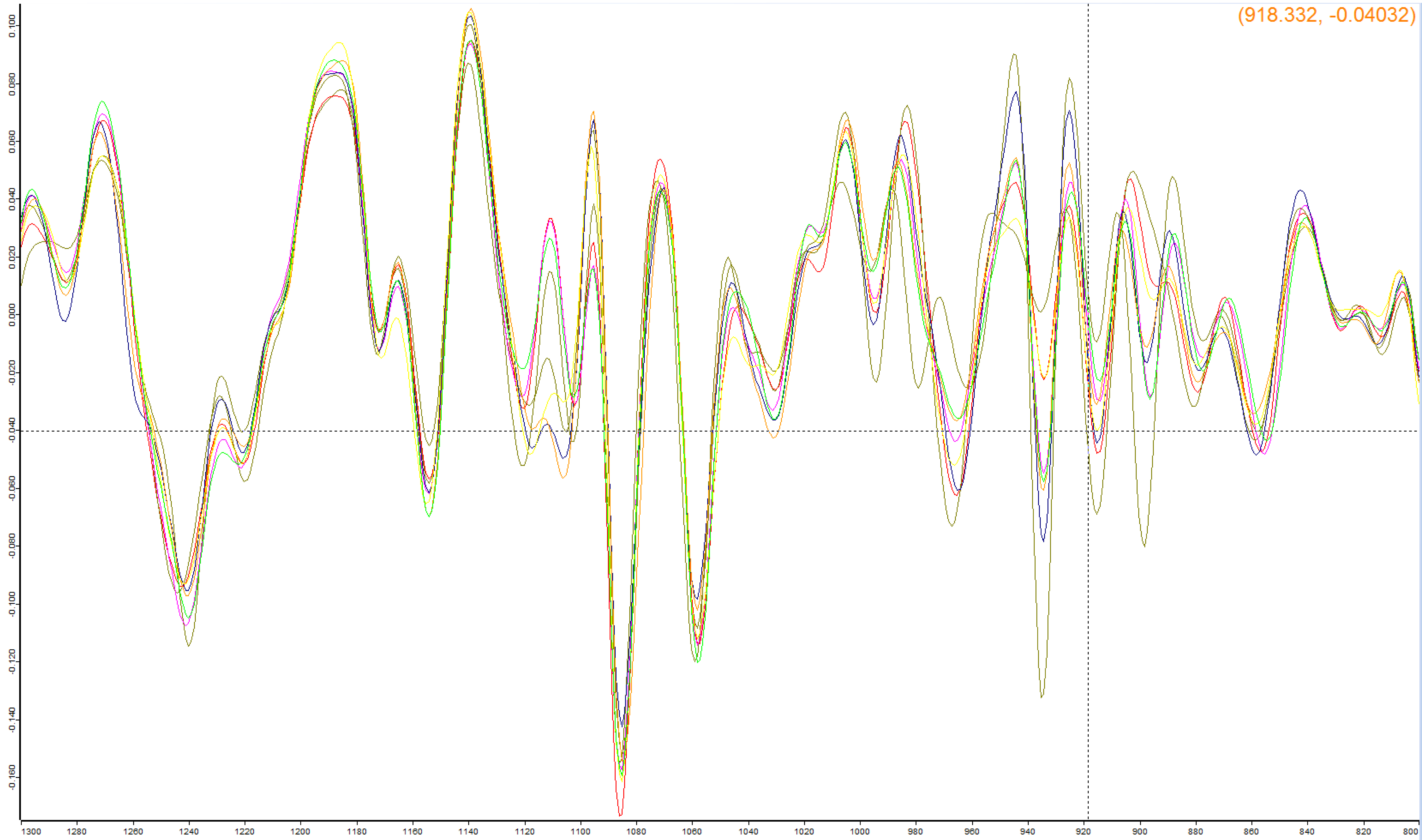

Supplementary Figure S9. Representative vector-normalized second-derivative spectra of *L. monocytogenes* strain LL195, generated using OPUS software, are presented for visualization. The strain was cultured at 37°C for 24 h on the following media: ALOA (green), Blood agar (red), Oxford agar (grey), Palcam agar (purple), BHI agar (brown), BHI broth (orange), TSA (yellow), and RAPID'L.mono agar (black). The spectral range displayed is 1300–800 cm^{-1}.

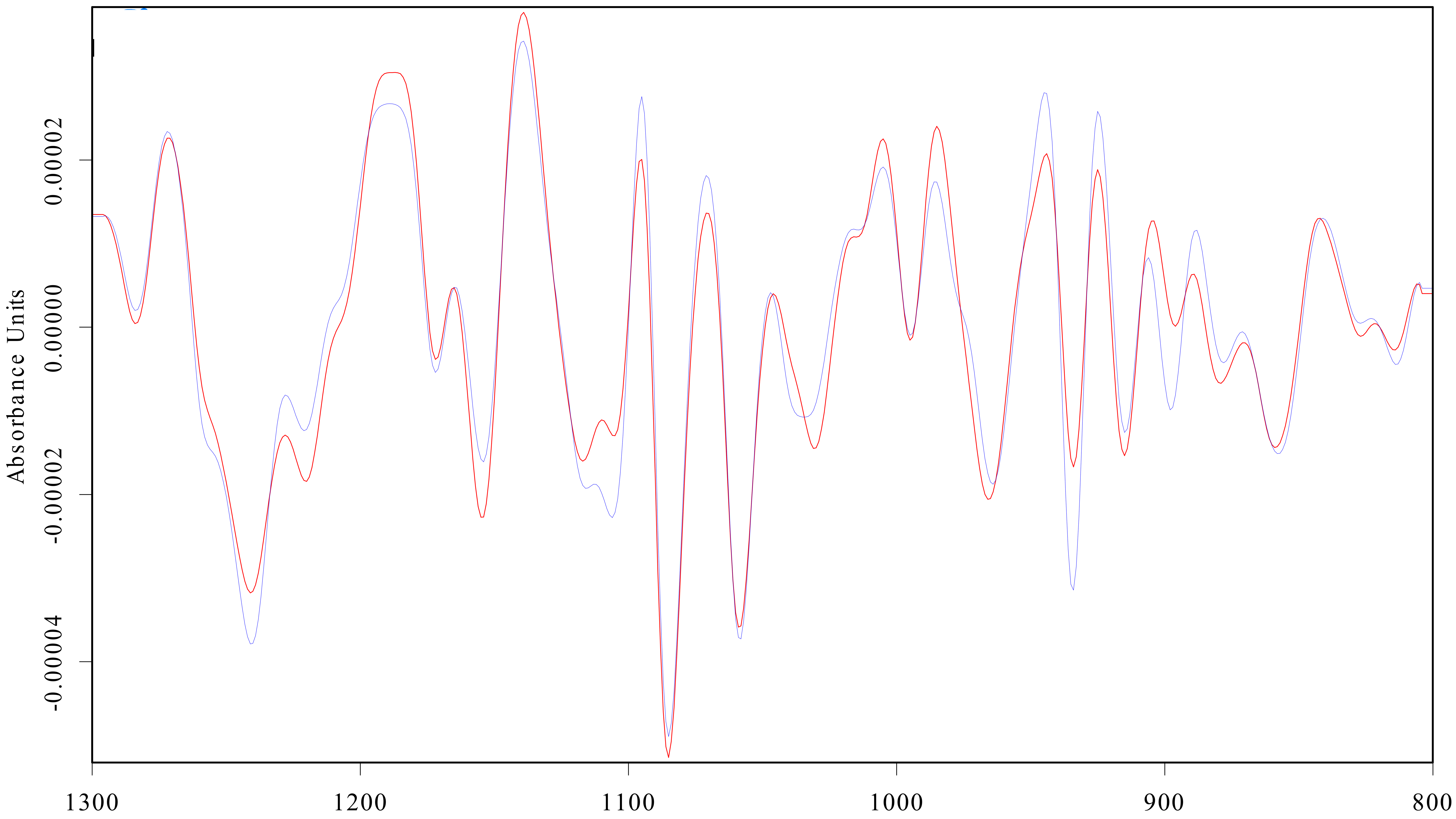

Supplementary Figure S10. Representative vector-normalized averaged second-derivative spectra of *L. monocytogenes* strain LL195, generated using OPUS software, are presented for visualization. The strain was cultured on Oxford agar at 25°C (blue) and 37°C (red) for 24 h. The spectral range displayed is 1300–800 cm^{-1}.

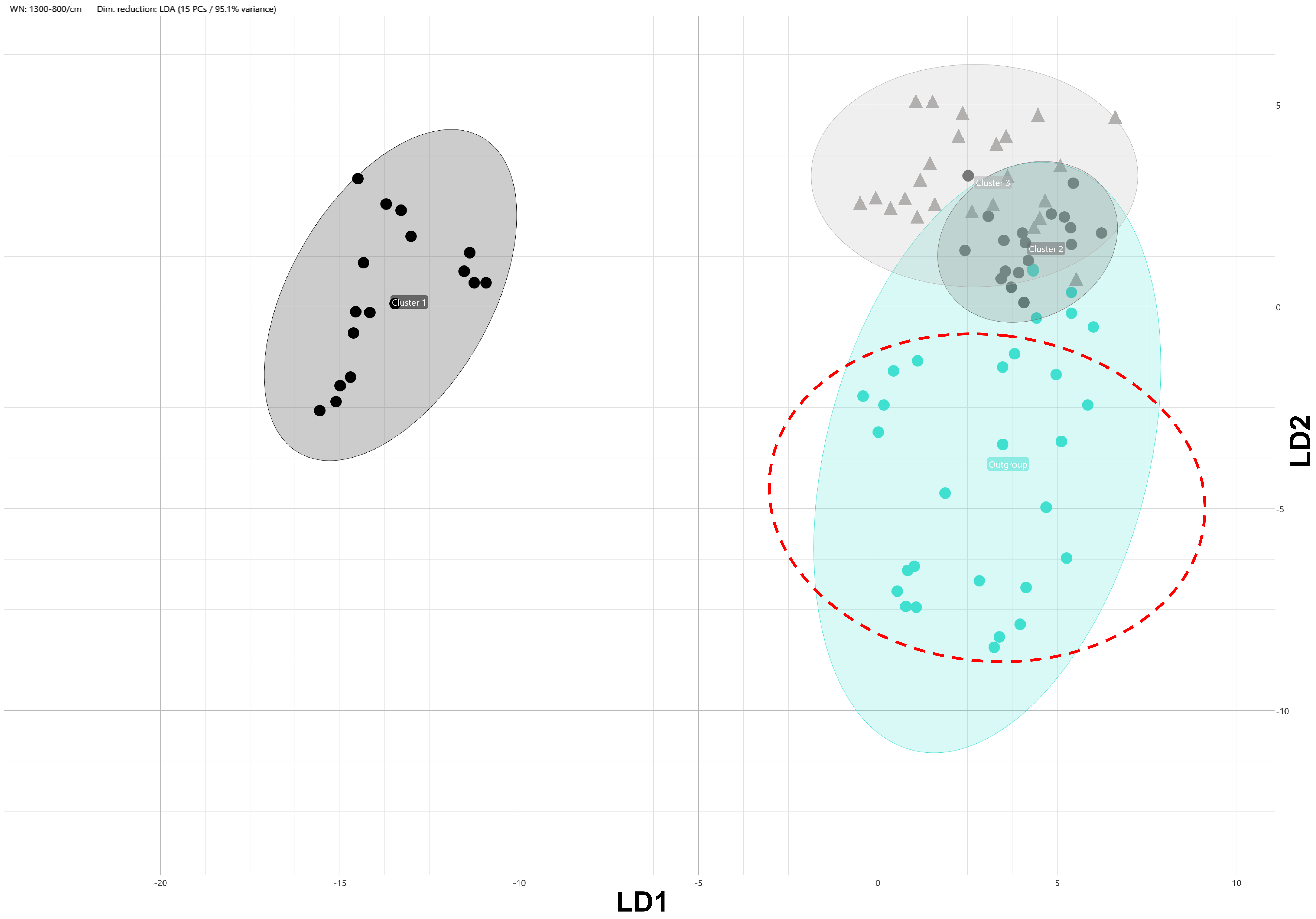

Supplementary Figure S11. Evaluation of FTIR as a real-time tool for cluster analysis. 2D scatter plot showing the FTIR spectra of *L. monocytogenes* CC9 strains from three cgMLST clusters and outgroup controls. Dotted red ellipse highlights isolates that can be excluded from sequencing. Spectra are color-coded by cluster, with each symbol (•) representing a technical replicate from at least two biological repeats. Dimensionality reduction using LDA with 15 principal components (PCs), 95.1% variance. The plot displays the first two LD axes.
